# Selectively anchoring single atoms on specific sites of supports for improved oxygen evolution

**DOI:** 10.1038/s41467-022-30148-3

**Published:** 2022-05-05

**Authors:** Zhirong Zhang, Chen Feng, Dongdi Wang, Shiming Zhou, Ruyang Wang, Sunpei Hu, Hongliang Li, Ming Zuo, Yuan Kong, Jun Bao, Jie Zeng

**Affiliations:** 1 Hefei National Research Center for Physical Sciences at the Microscale, Key Laboratory of Strongly-Coupled Quantum Matter Physics of Chinese Academy of Sciences, University of Science and Technology of China, Hefei, Anhui 230026 P. R. China; 2 National Synchrotron Radiation Laboratory, University of Science and Technology of China, Hefei, Anhui 230026 P. R. China; 3 Key Laboratory of Surface and Interface Chemistry and Energy Catalysis of Anhui Higher Education Institutes, Department of Chemical Physics, University of Science and Technology of China, Hefei, Anhui 230026 P. R. China

**Keywords:** Catalyst synthesis, Electrocatalysis, Electrocatalysis, Nanoscale materials, Electrocatalysis

## Abstract

The homogeneity of single-atom catalysts is only to the first-order approximation when all isolated metal centers interact identically with the support. Since the realistic support with various topologies or defects offers diverse coordination environments, realizing real homogeneity requires precise control over the anchoring sites. In this work, we selectively anchor Ir single atoms onto the three-fold hollow sites (Ir_1_/T_O_–CoOOH) and oxygen vacancies (Ir_1_/V_O_–CoOOH) on defective CoOOH surface to investigate how the anchoring sites modulate catalytic performance. The oxygen evolution activities of Ir_1_/T_O_–CoOOH and Ir_1_/V_O_–CoOOH are improved relative to CoOOH through different mechanisms. For Ir_1_/T_O_–CoOOH, the strong electronic interaction between single-atom Ir and the support modifies the electronic structure of the active center for stronger electronic affinity to intermediates. For Ir_1_/V_O_–CoOOH, a hydrogen bonding is formed between the coordinated oxygen of single-atom Ir center and the oxygenated intermediates, which stabilizes the intermediates and lowers the energy barrier of the rate-determining step.

## Abstract (web summary)

While single-atom catalysts offer well-defined structures, the homogeneity of the active sites is determined by localized coordination environments. Here, authors anchor Ir single atoms onto different sites on CoOOH and show how their distinct coordinations activate oxygen-evolving electrocatalysis

## Introduction

Single-atom catalysts (SACs), defined as spatially isolated metal atoms anchored on supports, have exhibited potential applications in various energy-conversion reactions^1–6^. Apart from their maximized atomic utilization, SACs are famous for their uniform active sites which benefit the selectivity and offer an ideal platform for mechanistic investigation^7–10^. However, the homogeneity of SACs is only to the first order approximation when all isolated metal centers interact identically with the support, because their catalytic performance is not only determined by the central metal species but also influenced by the surrounding coordinated atoms^11–14^. For instance, single-atom Fe coordinated by pyrrolic and pyridinic nitrogen of nitrogen-doped carbon gave rise to different oxidation states of +3 and +2, respectively, resulting in differentiated adsorption to CO_2_^15^. When Pt single atoms were coordinated with N and Mo atoms, the Pt-N bond was more favorable for the depletion of Pt *d*-band than Pt-Mo bond, leading to weaker adsorption to oxygen reduction intermediates^16^. Therefore, achieving real homogeneity of SACs requires precise control over the coordination environment of each metal center.

**Fig. 1:**
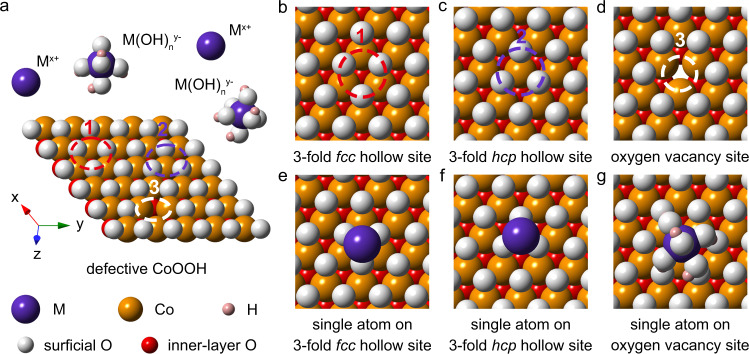
Scheme of defective CoOOH surface for anchoring single atoms in alkaline solutions. **a** Oxygen-terminated (001) surface of defective CoOOH with oxygen vacancies. **b**–**d** Potential anchoring sites on defective CoOOH, including three-fold (3-fold) *fcc* hollow site (**b**), 3-fold *hcp* hollow site (**c**), and oxygen vacancy site (**d**). **e**–**g** Single atoms anchored on different surface sites of defective CoOOH. In the aqueous synthesis of SACs, the metal ions are anchored onto the support through electrostatic adsorption. When using an alkaline solution, the metal precursors form both metal cations (M^x+^) and metal hydroxyl anions (M(OH)_n_^y−^). Due to their different electronegativities, the positive M^x+^ ions are inclined to adsorb on three-fold hollow sites (**e**, **f**), while M(OH)_n_^y−^ ions are prone to anchor onto oxygen vacancy sites (**g**).

It is challenging to realize identical active sites in SACs since the realistic support surface with various topologies or defects has different anchoring sites and accordingly diverse coordination environments. Taking CoOOH as an example, the perfect (001) surface of CoOOH is terminated with oxygen atoms, whereas the synthetic process usually induces oxygen vacancies on the surface (Fig. 1a). When the defective CoOOH is utilized as the support for anchoring single atoms, it offers different possible sites to immobilize the metal species, including the three-fold facial center cubic (*fcc*) hollow site of oxygen (Fig. 1b), three-fold hexagonal close packed (*hcp*) hollow site of oxygen (Fig. 1c), and oxygen vacancy site (Fig. 1d). In the aqueous synthesis of SACs, the metal ions are anchored onto the support through electrostatic adsorption^17,18^. When using an alkaline solution, the metal precursors form both metal cations (M^x+^) and metal hydroxyl anions (M(OH)_n_^y-^). Due to their different electronegativities, the positive M^x+^ ions are inclined to adsorb on three-fold hollow sites (Fig. 1e, f), while M(OH)_n_^y-^ ions are prone to anchor onto oxygen vacancy sites (Fig. 1g). If an electric field is applied, the positive or negative potential can drive a certain type of ions to deposit at unitary anchoring sites on the support. Therefore, electrochemical deposition method shows great potential to precisely anchor SACs on specific sites of supports.

In this work, we selectively anchor Ir single atoms on specific sites of defective CoOOH support via an electrochemical deposition method. Through cathodic electrochemical deposition, singly-dispersed iridium cations are deposited onto the three-fold hollow sites of surficial oxygen (Ir_1_/T_O_–CoOOH), while anodic electrochemical deposition drives isolated iridium anions to locate at oxygen vacancies (Ir_1_/V_O_–CoOOH). The impact of different anchoring sites on catalytic performance is probed in oxygen evolution reaction (OER). Specifically, Ir_1_/V_O_–CoOOH exhibits an OER overpotential of 200 mV at 10 mA cm^−2^, which is 70 mV lower than that of Ir_1_/T_O_–CoOOH. Spectroscopic measurements and density functional theory calculations reveal that single atoms on the two anchoring sites activate CoOOH through different mechanisms. For Ir_1_/T_O_–CoOOH, the band gap is decreased due to electron transfer from CoOOH to single-atom Ir, leading to stronger electronic affinity to intermediates than original CoOOH. For Ir_1_/V_O_–CoOOH, the single-atom Ir interacts with oxygenated intermediates through a configural effect rather than electronic adsorption tuning. A hydrogen bonding is formed between the coordinated oxygen of single-atom Ir center and the oxygenated intermediates, which stabilizes the intermediates and lowers the energy barrier of the rate-determining step.

## Results

### Synthesis and characterizations

We first synthesized the CoOOH support through a co-precipitation method^19^. The as-obtained CoOOH displayed a hexagonal nanosheet morphology in the transmission electron microscopy (TEM) image (Supplementary Fig. 1a). Energy-dispersive X-ray (EDX) elemental mapping image revealed the uniform distribution of Co and O elements in the material (Supplementary Fig. 1b). In the X-ray diffraction (XRD) pattern, three characteristic peaks were located at 2*θ* = 20.1^o^, 38.9^o^, and 50.5^o^, coinciding with the (003), (012), and (015) planes of CoOOH, respectively (PDF #07-0169) (Supplementary Fig. 1c). Electron spin resonance (ESR) spectrum and X-ray photoelectron spectroscopy (XPS) measurements were also performed to characterize the CoOOH surface. An ESR signal was detected at *g* = 2.01, suggesting the presence of oxygen vacancies on CoOOH^20^ (Supplementary Fig. 1d). The O 1 *s* XPS spectrum was deconvoluted into four peaks at 532.8, 531.7, 530.8, and 529.7 eV, corresponding to adsorbed H_2_O, oxygen vacancies, Co-OH, and Co–O, respectively^21^ (Supplementary Fig. 1e). In the deconvoluted Co 2*p* XPS spectrum, the peaks at 780.3 and 795.4 eV were attributed to Co^3+^ 2*p*_3/2_ and Co^3+^ 2*p*_1/2_, respectively, confirming the presence of Co^3+^ (Supplementary Fig. 1f)^21^. While the Co^2+^ 2*p*_3/2_ peak at 781.7 eV and Co^2+^ 2*p*_1/2_ peak at 797.1 eV, together with their satellite peaks at 790.2 and 805.6 eV, validated the presence of Co^2+^ (Supplementary Fig. 1f)^21^. The co-presence of Co^3+^ and Co^2+^ suggested that the valence state of Co was lower than +3, which validated the existence of oxygen vacancies on CoOOH surface^21^. The above results collectively demonstrated that the surface of CoOOH nanosheets was not uniform with both ordered atomic arrangement and defective sites, which provides distinct anchoring sites for single atoms.

**Fig. 2:**
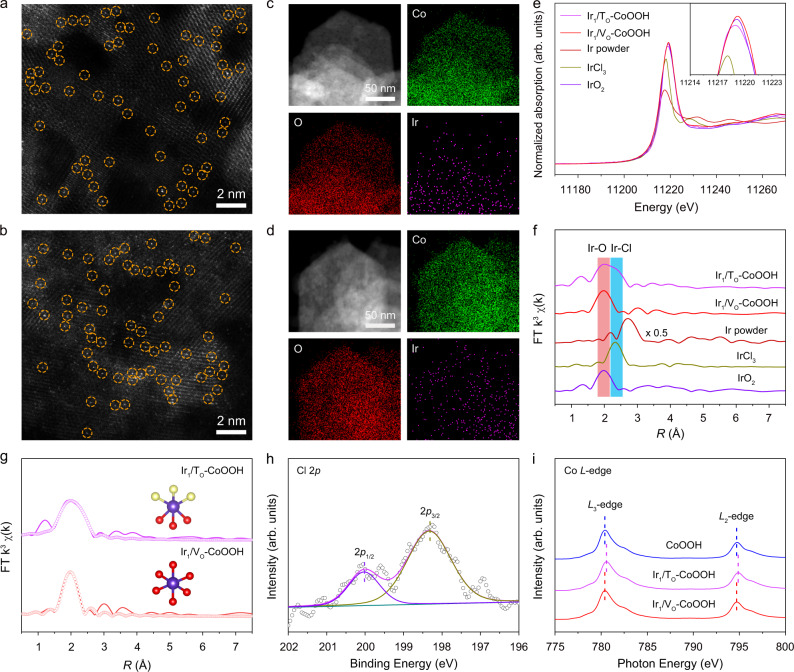
Structural characterizations of Ir single atoms on CoOOH nanosheets. **a**, **b** HAADF-STEM images of Ir_1_/T_O_–CoOOH (**a**) and Ir_1_/V_O_–CoOOH (**b**). Singly-dispersed Ir atoms are indicated by yellow circles. **c**, **d** EDX elemental mapping of Ir_1_/T_O_–CoOOH (**c**) and Ir_1_/V_O_–CoOOH (**d**). **e**, **f** Normalized XANES (**e**) and EXAFS (**f**) spectra at the Ir *L*_3_-edge for Ir_1_/T_O_–CoOOH and Ir_1_/V_O_–CoOOH. Ir powder, IrCl_3_, and IrO_2_ were used as references. **g** Experimental and fitting EXAFS results of Ir_1_/T_O_–CoOOH and Ir_1_/V_O_–CoOOH. The experimental and fitting results are shown in solid lines and circles, respectively. The inset atomic models are the first-shell coordination of Ir. The red, yellow, and purple spheres represent O, Cl, and Ir atoms, respectively. **h** Cl 2*p* XPS spectrum of Ir_1_/T_O_–CoOOH. **i** Co *L*-edge XAS spectra of CoOOH, Ir_1_/T_O_–CoOOH, and Ir_1_/V_O_–CoOOH.

Next, we selectively anchored Ir single atoms onto specific sites of defective CoOOH surface by an electrochemical deposition method. Through cathodic and anodic deposition, we obtained Ir_1_/T_O_–CoOOH and Ir_1_/V_O_–CoOOH, respectively. No noticeable metal or metal oxide particles were observed in the TEM images of Ir_1_/T_O_–CoOOH and Ir_1_/V_O_–CoOOH (Supplementary Fig. 2a, b). The side views of these nanosheets showed a thickness of 1.33 nm with four Co layers (Supplementary Fig. 3). The interspace between the layers was 0.445 nm, corresponding to the (003) plane of CoOOH (Supplementary Fig. 3). This result accorded with the XRD patterns with a major characteristic peak of the (003) plane at 2*θ* = 20.1^o^, while minor peaks attributing to (012) and (015) planes of CoOOH were related to the exposed edges (Supplementary Fig. 2c, d). Aberration-corrected high-angle annular dark-field scanning TEM (HAADF-STEM) images further revealed the atomic dispersion of Ir atoms on the CoOOH support for both samples (Fig. 2a, b). In addition, EDX elemental mapping image showed the uniform distribution of Ir element across the support (Fig. 2c, d). In order to identify the anchoring sites of single atoms, we fixed the projection direction of the HAADF-STEM images to [−111] of CoOOH. For Ir_1_/T_O_–CoOOH, the bright Ir atoms were located at ordered lattice sites, which were ascribed to the three-fold hollow sites in the simulated HAADF-STEM image (Supplementary Fig. 4). For Ir_1_/V_O_–CoOOH, single Ir atoms were discerned in the interstice of three triangular lattice sites, corresponding to the oxygen vacancy sites as indicated by the simulated HAADF-STEM image (Supplementary Fig. 4). The mass loadings of Ir in Ir_1_/T_O_–CoOOH and Ir_1_/V_O_–CoOOH were determined to be 1.2% and 1.3%, respectively, by inductively coupled plasma-atomic emission spectroscopy (ICP-AES).

We further investigated the electronic structure and coordinated environment of Ir_1_/T_O_–CoOOH and Ir_1_/V_O_–CoOOH by X-ray absorption near-edge spectroscopy (XANES) and extended X-ray absorption fine structure (EXAFS) measurements. Figure 2e showed the Ir *L*_3_-edge XANES spectra of Ir_1_/T_O_–CoOOH and Ir_1_/V_O_–CoOOH as referred to Ir powder, IrCl_3_, and IrO_2_. The white line intensity indicated that the valence state of Ir in Ir_1_/T_O_–CoOOH was between +3 and +4, while the valence state of Ir in Ir_1_/V_O_–CoOOH was slightly higher than +4. The difference in Ir valence states may relate to transfer of electrons under reductive or oxidative conditions in their synthesis. In the EXAFS spectra, neither Ir_1_/T_O_–CoOOH nor Ir_1_/V_O_–CoOOH showed a peak at 2.71 Å as referred to Ir powder, indicating the absence of Ir–Ir bonding (Fig. 2f). Thus the isolated atomic distribution of Ir species on CoOOH was validated for both samples. Ir_1_/T_O_–CoOOH showed two peaks at 1.97 and 2.32 Å, which were assigned to Ir–O and Ir–Cl bonding, respectively (Fig. 2f). The fitting result suggested that Ir_1_/T_O_–CoOOH had an Ir–O and Ir–Cl coordination number of 3.3 and 3.2, respectively, forming a full coordination of IrCl_3_O_3_ (Fig. 2g and Supplementary Table 1). The presence of Cl in Ir_1_/T_O_–CoOOH was also affirmed by the XPS spectrum with Cl 2*p*_1/2_ and 2*p*_3/2_ peaks at 200.0 and 198.3 eV, respectively, which is most likely to stem from the depositing species using IrCl_4_ precursors (Fig. 2h). In comparison, the EXAFS spectrum of Ir_1_/V_O_–CoOOH only exhibited a prominent Ir–O peak at 2.01 Å (Fig. 2f). The coordination number of Ir–O bonding was fitted to be 5.9, suggesting a coordination of IrO_6_ for Ir_1_/V_O_–CoOOH (Fig. 2g and Supplementary Table 1). In addition, the absence of first-shell Co-Ir scattering excluded the direct binding of Ir single atoms to Co sites for both Ir_1_/T_O_–CoOOH and Ir_1_/V_O_–CoOOH, indicating that Ir species were connected to the support through Ir–O bonding.

Based on the above experimental results, we speculated the mechanism behind selectively anchoring Ir single atoms on specific sites of the CoOOH surface. For Ir_1_/T_O_–CoOOH obtained from cathodic deposition, the negative electronic field drove iridium-based cations to deposit onto the electrode. The depositing species should be IrCl_3_^+^ cations as inferred from the first-shell coordination of IrCl_3_O_3_. Since there are three possible anchoring sites on CoOOH, density functional theory (DFT) calculations were conducted to estimate the formation energy of IrCl_3_^+^ on the sites in Fig. 1b–d. IrCl^3+^ species displayed a formation energy of −0.86 eV on three-fold *fcc* hollow site, which was 0.46 eV and 2.74 lower than those on three-fold *hcp* hollow site and oxygen vacancy site, respectively (Supplementary Fig. 5). The higher formation energy of IrCl_3_^+^ on the *hcp* site may result from the closer distance of inner-layer Co atom to Ir center, where the electrostatic repulsion from Co led to lower stability. Thus, Ir atoms of Ir_1_/T_O_–CoOOH were the most stable and likely to anchor on three-fold *fcc* hollow sites, where the surficial oxygen atoms provided lone-pair electrons and negative charge to combine IrCl_3_^+^ cations. For Ir_1_/V_O_–CoOOH synthesized by anodic deposition, the positive electronic field led to iridium-based anions to deposit onto the electrode. The deposited species should be Ir(OH)_6_^2-^ anions with a full coordination of IrO_6_ (ref. ^22,23^). The stability of Ir(OH)_6_^2-^ on three-fold hollow sites and oxygen vacancy site were compared by DFT calculations. The formation energy of Ir(OH)_6_^2-^ on oxygen vacancy site (−1.44 eV) was significantly lower than those on three-fold *fcc* (1.13 eV) and *hcp* (1.06 eV) hollow site, proving the tendency of Ir(OH)_6_^2−^ anchoring on oxygen vacancies (Supplementary Fig. 6). As a result, the Ir(OH)_6_^2−^ anions were prone to bind onto electron-accepting oxygen vacancy sites, where the deficiency of oxygen gave rise to localized positive charge to attract anions. In this process, the Ir(OH)_6_^2−^ anions are deprotonated under the oxidative potential, leading to the Ir valence state of Ir_1_/V_O_–CoOOH higher than +4 due to loss of electrons.

To elucidate how the anchored single atoms influenced the support, we further performed X-ray absorption spectroscopy (XAS) analyses and XPS measurements. In the Co *L*-edge XAS spectrum of Ir_1_/T_O_–CoOOH, the Co *L*_3_- and *L*_2_-edge peaks were shifted to higher energy relative to CoOOH, indicating an increased valence state of Co (Fig. 2i). The increased Co valence state indicated an electron transfer from CoOOH to Ir single atoms due to metal-support interaction. For Ir_1_/V_O_–CoOOH, the Co *L*_3_- and *L*_2_-edge peaks displayed negligible change compared with CoOOH, suggesting an unchanged valence state of Co (Fig. 2i). Hence, the charge transfer between Co and Ir is relatively weak in Ir_1_/V_O_–CoOOH. The Co 2*p* XPS spectra also showed the increased valence state of Ir_1_/T_O_–CoOOH and the unvaried valence state of Ir_1_/V_O_–CoOOH (Supplementary Fig. 7). As revealed by O 1 *s* XPS spectra, the oxygen vacancy concentration of Ir_1_/V_O_–CoOOH decreased relative to CoOOH, while that of Ir_1_/T_O_–CoOOH showed an insignificant change after anchoring Ir single atoms (Supplementary Fig. 8). The results further implied that the Ir atomic species of Ir_1_/V_O_–CoOOH occupied part of the oxygen vacancy sites on CoOOH.

### Electrocatalytic performance towards oxygen evolution

**Fig. 3:**
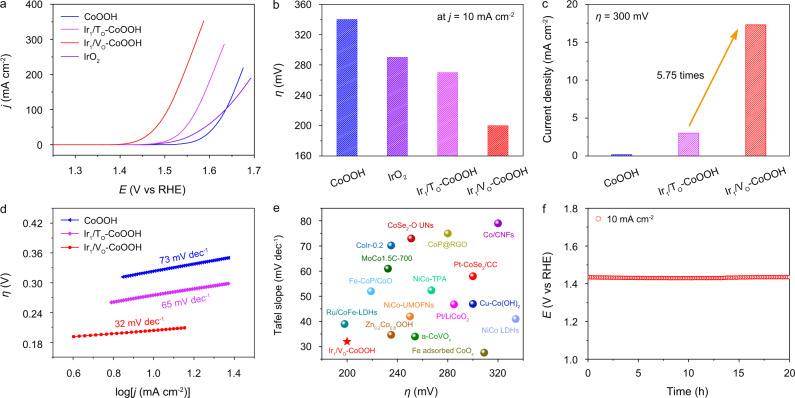
Electrocatalytic performance towards OER. **a** Polarization curves of CoOOH, Ir_1_/T_O_–CoOOH, and Ir_1_/V_O_–CoOOH. IrO_2_ was used as reference. The measurements were conducted in 1.0 M KOH. **b** Overpotentials at 10 mA cm^−2^ for CoOOH, Ir_1_/T_O_–CoOOH, and Ir_1_/V_O_–CoOOH. **c** Specific activities of CoOOH, Ir_1_/T_O_–CoOOH, and Ir_1_/V_O_–CoOOH normalized against ECSA at an overpotential of 300 mV. **d** Tafel slopes of CoOOH, Ir_1_/T_O_–CoOOH, and Ir_1_/V_O_–CoOOH. **e** Comparison of Tafel slopes and overpotentials at 10 mA cm^−2^ for currently reported cobalt-based OER catalysts. **f** Chronopotentiometric curves of Ir_1_/V_O_–CoOOH towards OER at 10 mA cm^−2^ for 20 h.

Inspired by the distinct geometric and electronic structures of site-selective Ir SACs, we further evaluated their catalytic performance towards oxygen evolution, one of the most essential reactions for energy conversion^24–28^. During synthesis, the alkaline environment played an essential role in forming the specific OH-coordinated structure of Ir_1_/V_O_–CoOOH. Moreover, to prevent the dissolution of the CoOOH substrate in acid, the electrochemical measurements were conducted in 1 M KOH electrolyte. As showed in the polarization curves, Ir_1_/T_O_–CoOOH and Ir_1_/V_O_–CoOOH showed dramatically improved activity relative to the original CoOOH, notably outperforming commercial IrO_2_ as well (Fig. 3a and Supplementary Fig. 9). Especially for Ir_1_/V_O_–CoOOH, the overpotential (*η*) required to reach a current density of 10 mA cm^−2^ was 200 mV, which was 70 mV lower than that of Ir_1_/T_O_–CoOOH (Fig. 3b). Besides, the Faradaic efficiencies were estimated to be over 99% for both samples, validating the exclusive contribution from OER to the current densities (Supplementary Fig. 10).

In order to compare the intrinsic activity of the samples, the current densities were normalized against their electrochemically active surface areas (ECSAs) (Supplementary Fig. 11). Ir_1_/V_O_–CoOOH showed a specific activity of 17.3 mA cm^−2^ at an overpotential of 300 mV, which was 5.75 times higher than that of Ir_1_/T_O_–CoOOH (Fig. 3c). The normalized mass activities against the mass of CoOOH demonstrated that Ir_1_/V_O_–CoOOH was the most active with a value of 605.4 A g^−1^_CoOOH_ at an overpotential of 300 mV, which was 5.84 times higher than that of Ir_1_/T_O_–CoOOH (Supplementary Fig. 12a, b). The normalized mass activity against active CoOOH adjacent to Ir of Ir_1_/V_O_–CoOOH was 5.40 times higher than that of Ir_1_/T_O_–CoOOH at *η* = 300 mV (Supplementary Fig. 12c, d). When normalized to Ir loadings, the mass activity of Ir_1_/V_O_–CoOOH was also 5.40 times higher than that of Ir_1_/T_O_–CoOOH at *η* = 300 mV, despite different values from those normalizing to active CoOOH (Supplementary Fig. 12e, f). Similarly, the turnover frequency (TOF) was first calculated based on the number of active Co sites. At an overpotential of 300 mV, Ir_1_/V_O_–CoOOH showed a higher TOF of 0.144 s^−1^, largely surpassing Ir_1_/T_O_–CoOOH with a TOF of 0.025 s^−1^ by 5.76 times (Supplementary Fig. 13a). When based on the number of Ir sites, the TOF of Ir_1_/V_O_–CoOOH was 5.39 times higher than that of Ir_1_/T_O_–CoOOH at *η* = 300 mV (Supplementary Fig. 13b). Due to the similar Ir mass loading of Ir_1_/T_O_–CoOOH and Ir_1_/V_O_–CoOOH, the relative mass activities and TOFs of Ir_1_/V_O_–CoOOH were superior than those of Ir_1_/T_O_–CoOOH when normalizing to either Co or Ir. The above results suggested that anchoring Ir single atoms onto oxygen vacancies showed superior OER activity than onto three-fold *fcc* hollow sites.

To preclude the influence of electrochemical treatments in the synthetic process, the OER performance of CoOOH treated by cathodic and anodic electrochemical methods without anchoring Ir single atoms was evaluated. The almost overlapped polarization curves indicated that these CoOOH had similar OER activities. Thus, the increments in current densities of Ir_1_/T_O_–CoOOH and Ir_1_/V_O_–CoOOH should all be attributed to anchored Ir single atoms (Supplementary Fig. 14).

The reaction kinetics was evaluated by calculating the Tafel slopes. CoOOH, Ir_1_/T_O_–CoOOH, and Ir_1_/V_O_–CoOOH showed decreasing Tafel slope values from 73, 65, to 32 mV dec^−1^, respectively (Fig. 3d). The lower Tafel slope value of Ir_1_/V_O_–CoOOH suggested its faster reaction kinetics relative to Ir_1_/T_O_–CoOOH. For comparison, both the overpotential at 10 mA cm^−2^ and the Tafel slope of Ir_1_/V_O_–CoOOH were lower than a number of reported Co-based OER catalysts (Fig. 3e and Supplementary Table 2). In addition, electrochemical impedance analysis demonstrated the interfacial charge-transfer resistance of the catalysts (Supplementary Fig. 15). The smaller semicircle diameter of Ir_1_/V_O_–CoOOH than that of Ir_1_/T_O_–CoOOH implied that Ir_1_/V_O_–CoOOH had faster charge transfer at the interface, contributing to its faster OER kinetics.

Durability tests of Ir_1_/V_O_–CoOOH with the superior activity were also conducted. During the galvanostatic test at 10 mA cm^−2^ for 20 h, the OER potential showed no noticeable decay (Fig. 3f). The morphology and electronic structures of the sample were characterized after the durability test. No obvious metal clusters or particles were identified in the XRD pattern and TEM image, while the Ir atoms still showed isolated dispersion on the support in the HAADF-STEM image, confirming that Ir_1_/V_O_–CoOOH maintained its atomic structure after the test (Supplementary Fig. 16). The Ir mass loading of Ir_1_/Vo–CoOOH after the durability test was measured to be 1.29% by ICP-AES, which was close to that (1.3%) before and suggested no notable leaching of single atoms. As revealed by the Co 2*p* XPS spectrum, the valence state of Co in Ir_1_/V_O_–CoOOH after the durability test showed negligible change compared with that before the test (Supplementary Fig. 16).

### In-situ spectroscopic characterizations

**Fig. 4:**
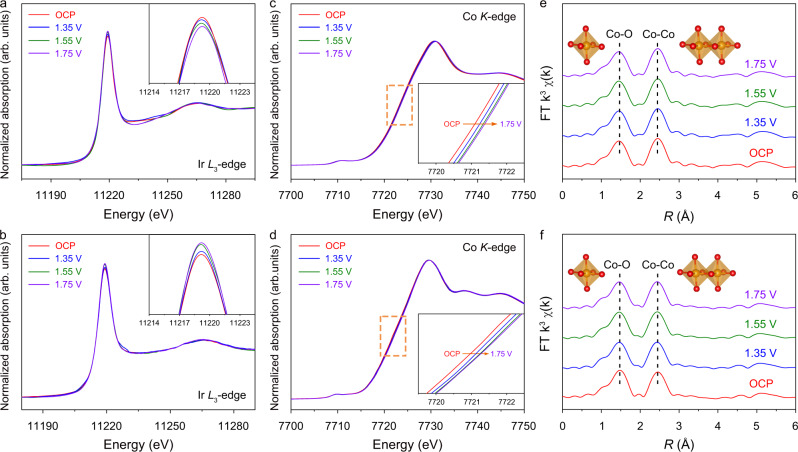
In-situ spectroscopic characterizations of Ir_1_/T_O_–CoOOH (top) and Ir_1_/V_O_–CoOOH (bottom). **a**, **b** In-situ Ir *L*_3_-edge XANES spectra of Ir_1_/T_O_–CoOOH (**a**) and Ir_1_/V_O_–CoOOH (**b**) at different applied potentials. The inset is the magnified spectra at the white line peak. **c**, **d** In-situ Co *K*-edge XANES spectra of Ir_1_/T_O_–CoOOH (**c**) and Ir_1_/V_O_–CoOOH (**d**) at different applied potentials. The inset is the magnified spectra at the adsorption edge. **e**, **f** In-situ Co *K*-edge EXAFS spectra of Ir_1_/T_O_–CoOOH (**e**) and Ir_1_/V_O_–CoOOH (**f**) at different applied potentials.

We employed a series of in-situ spectroscopic measurements to probe the structural changes of Ir_1_/T_O_–CoOOH and Ir_1_/V_O_–CoOOH under the realistic OER conditions. The in-situ Ir *L*_3_-edge XANES spectra of both Ir_1_/T_O_–CoOOH and Ir_1_/V_O_–CoOOH showed increasing white line intensity from open circuit potential (OCP) to 1.75 V, corresponding to an increased valence state of Ir under higher applied potentials (Fig. 4a, b). The in-situ Co *K*-edge positions were also shifted to higher energy due to increased valence state of Co with increasing applied potentials (Fig. 4c, d). The valence state of both Co and Ir in Ir_1_/T_O_–CoOOH and Ir_1_/V_O_–CoOOH increased under the applied potential, manifesting their participations in the OER process with electron transfer. Nevertheless, the energy shift of Co *K*-edge position from OCP to 1.75 V was less than 0.5 eV, suggesting the high stability of the material under oxidative potentials. Moreover, according to the in-situ Co *K*-edge EXAFS spectra of Ir_1_/T_O_–CoOOH and Ir_1_/V_O_–CoOOH, the peaks ascribing to Co–O and Co–Co bonds remained at 1.46 and 2.44 Å, respectively, when the applied potential was increased from OCP to 1.75 V (Fig. 4e, f). The results validated no noticeable structural change of Ir_1_/T_O_–CoOOH and Ir_1_/V_O_–CoOOH under the oxidative potentials. In the in-situ Raman spectroscopy of Ir_1_/V_O_–CoOOH, a peak was observed at 499 cm^−1^ for all potentials (Supplementary Fig. 17). The peak was assigned to the *E*_g_ vibration mode of Co–O in CoOOH moieties, being a characteristic feature for CoOOH phase^29^. The invariable peak position at different potentials further confirmed CoOOH as the stable active phase.

### Mechanistic studies

**Fig. 5:**
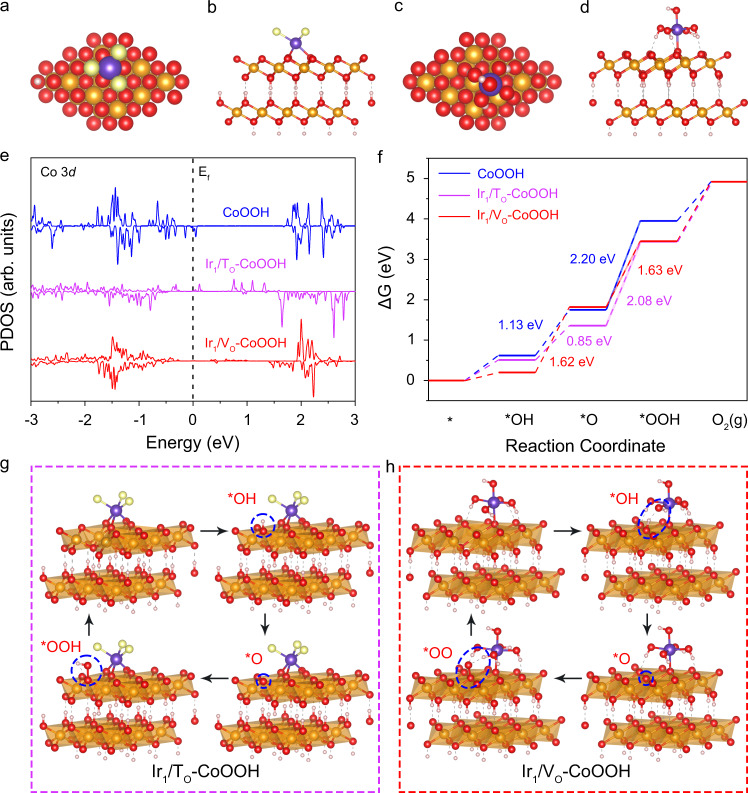
OER mechanism studies for Ir_1_/T_O_–CoOOH and Ir_1_/V_O_–CoOOH. **a**, **b** Schematic structure model of Ir_1_/T_O_–CoOOH from top (**a**) and side (**b**) views. **c**, **d** Schematic atomic structure of Ir_1_/V_O_–CoOOH from top (**c**) and side (**d**) views. **e** Co 3*d* projected density of states (PDOS) in CoOOH, Ir_1_/T_O_–CoOOH, and Ir_1_/V_O_–CoOOH. **f**, Free energy diagrams. **g**, **h** The schematic OER pathway for Ir_1_/T_O_–CoOOH (**g**) and Ir_1_/V_O_–CoOOH (**h**). The pink, red, yellow, orange, and purple spheres represent H, O, Cl, Co, and Ir atoms, respectively. The dashed grey line indicates the hydrogen bonding. The reaction intermediates are indicated by blue circles.

To investigate the detailed OER mechanism on both samples, we carried out DFT calculations to elucidate the electronic structures. The atom models of single IrCl_3_^+^ and Ir(OH)_6_^2-^ on a two-layer 3 × 3 supercell of CoOOH (001) were firstly established for Ir_1_/T_O_–CoOOH and Ir_1_/V_O_–CoOOH, respectively (Fig. 5 and Supplementary Fig. 18). As demonstrated in previous DFT calculations, the Ir species of Ir_1_/T_O_–CoOOH and Ir_1_/V_O_–CoOOH were the most stable at three-fold *fcc* hollow sites and oxygen vacancy sites, respectively (Supplementary Fig. 5 and Supplementary Fig. 6). For Ir_1_/T_O_–CoOOH, the single-atom Ir was anchored onto the three-fold *fcc* hollow site with three dangling chlorine atoms (Fig. 5a, b). The post-moterm measurements showed that Ir_1_/T_O_–CoOOH maintained this coordination after the OER process (Supplementary Fig. 19). For Ir_1_/V_O_–CoOOH, one apex oxygen of Ir(OH)_6_^2-^ octahedra was fitted into the oxygen vacancy site (Fig. 5c). Simultaneously, four side OH of the octahedra formed hydrogen bonding with adjacent oxygen atoms on the CoOOH surface to stabilize the structure (Fig. 5d). Then the projected density of state (PDOS) analyses were conducted to elucidate the electronic structure based on the above atomic structures. Compared with CoOOH, edge states emerged at around the Fermi level in the Co 3*d* PDOS of Ir_1_/T_O_–CoOOH and diminished the band gap from 1.8 to 0.8 eV (Fig. 5e). The narrowed band gap benefits faster electron transfer from valence band maximum to conduction band minimum, which facilitates the reaction kinetics^30^. However, the band gap of Ir_1_/V_O_–CoOOH was the same as that (1.8 eV) of CoOOH, indicating that the improved OER performance was not originated from modified electronic structures but other causes (Fig. 5e).

The free energy diagram and reaction pathway were further calculated. Previous experimental and theoretical studies have suggested that OER on Ir-based catalysts requires two adjacent sites to proceed^31–34^. Mononuclear Ir sites are generally inactive with large overpotential to drive^34^. To further illustrate the OER activity of Ir single atoms, we anchored them on OER-inert nitrogen-doped carbon (N–C) using the same synthetic methods as for Ir_1_/T_O_–CoOOH and Ir_1_/V_O_–CoOOH (Supplementary Fig. 20 and Supplementary Fig. 21). The Ir single atoms on N–C showed insignificant enhancements in current densities relative to pure N–C, indicating the inertness of single Ir atoms towards OER (Supplementary Fig. 22). Therefore, Co atoms were chosen as the active site for the adsorption of intermediates following the conventional adsorbate evolution mechanism. The reaction started from the adsorption of OH^−^ ion, followed by the sequential deprotonation to form O*, O–O bonding formation to generate OOH*, and desorption to produce oxygen. The rate-determining step (RDS) at U = 0 V of all samples was the O–O bonding formation from O* to OOH* (Fig. 5f). For CoOOH, the energy barrier of the RDS was 2.20 eV. The RDS energy barrier of Ir_1_/T_O_–CoOOH was lowered to 2.08 eV, while that of Ir_1_/V_O_–CoOOH was decreased to a much lower value of 1.63 eV. The OER performance under different applied potentials was also calculated, which showed a linear correlation between the equilibrium potential and applied potential (Supplementary Fig. 23a). At U = 1.23 V, the RDS was still the formation of *OOH with an identical theoretical overpotential of 0.40 eV to that at U = 0 V, suggesting that the OER activity is independent of applied potentials (Supplementary Fig. 23b). Compared with other possible reaction mechanisms, the energy barriers of both Ir_1_/T_O_–CoOOH and Ir_1_/V_O_–CoOOH under this pathway on Co sites were the lowest (Supplementary Fig. 24 and Supplementary Fig. 25). Of note, the calculated energy barrier was from a thermodynamic view, which regarded potential-determining step as equivalent to RDS^35,36^. More precise estimation of the kinetic activation barrier needs to develop calculation methods to go beyond the thermodynamic overpotential.

In addition, the influences of surface coverages of Ir single atoms on the OER performance were probed. We expanded the calculation model to a two-layered 4*4 supercell with less coverage of Ir single atoms on CoOOH (Supplementary Fig. 26). The energy barriers of the RDS were 2.02 and 1.68 eV for Ir_1_/T_O_–CoOOH and Ir_1_/V_O_–CoOOH, respectively. The trivial changes within 0.05 eV relative to those under the 3*3 calculation model were within the numerical error caused by increased complexity of the calculation system.

Furthermore, we calculated the Gibbs free energy of the key intermediates. The adsorption mode of oxygenated intermediates on Ir_1_/T_O_–CoOOH was the same as that on CoOOH (Fig. 5g and Supplementary Fig. 27). Nevertheless, the free energies of *OH, *O, and *OOH on Ir_1_/T_O_–CoOOH were lower than those on CoOOH (Supplementary Table 3). The lower free energies are associated with stronger adsorption to the oxygenated species on Ir_1_/T_O_–CoOOH, which was resulted from the stronger electron affinities of Co with higher valence state than the original CoOOH^37^. Despite the enhanced adsorption of intermediates, the adsorption on Ir_1_/T_O_–CoOOH was too strong to reach an optimal energy barrier^38^. By contrast, though the free energy of *O (1.82 eV) on Ir_1_/V_O_–CoOOH was close to that (1.75 eV) on CoOOH, the free energies of *OH and *OOH was significantly lowered. As further revealed by the detailed reaction pathways, a hydrogen bonding was formed between *OH/*OOH and the coordinated oxygen of single-atom Ir center (Ir(OH)_6_) (Fig. 5h). Hence, the Ir species on CoOOH oxygen vacancies stabilized *OH and *OOH intermediates, leading to a near-optimal energy barrier. Moreover, the hydrogen bonding between *OOH and Ir(OH)_6_ was strong enough to pull the hydrogen from *OOH to adsorb on Ir(OH)_6_, forming a *OO intermediate. The *OO intermediate was further stabilized by the adsorbed H on Ir(OH)_6_ via hydrogen bonding. The deprotonated *OO was more stable with a lower Gibbs free energy of 3.45 eV than that of 3.69 eV for *OOH without deprotonation (Supplementary Fig. 28). In addition, the deprotonation of *OOH intermediate to *OO suggested a facilitated proton transfer at the presence of hydrogen bonding, which was in consistence with the Tafel measurements. Above all, anchoring single atoms onto the oxygen vacancy of CoOOH support improves the OER activity through the configural interaction between single-atom Ir and reaction intermediates.

## Discussion

On the basis of the above results, we can conclude that CoOOH was activated by Ir single atoms at different anchoring sites. These Ir single atoms with distinct coordination environments led to diverse mechanisms to tune the OER performance. For Ir_1_/T_O_–CoOOH, the improved OER activity was originated from the overall electronic effect after introducing single atoms. The strong metal-support interaction led to charge transfer from Co to Ir, which decreased the band gap for faster charge transfer and synchronously strengthened the adsorption of all reaction intermediates. In contrast, the metal-support interaction in Ir_1_/V_O_–CoOOH was not strong enough to modify the electronic structure of Co centers. However, the oxygen-coordinated Ir centers provided extra force to stabilize *OH and *OO intermediates on Co through hydrogen bonding, adjusting the energy barrier of RDS to the optimal value.

In this work, we report the site-selective anchoring of Ir single-atoms on defective CoOOH support via cathodic and anodic electrochemical deposition. Through cathodic deposition, IrCl_3_^+^ cations are deposited onto the electron-donating three-fold *fcc* hollow sites (Ir_1_/T_O_–CoOOH). While in anodic deposition, the positive electric field drives Ir(OH)_6_^2-^ anions to deposit onto electron-accepting oxygen vacancies (Ir_1_/V_O_–CoOOH). Ir_1_/T_O_–CoOOH and Ir_1_/V_O_–CoOOH both show improved oxygen evolution activity relative to CoOOH. For Ir_1_/T_O_–CoOOH, the increased activity is attributed to the decreased band gap and the strengthened electronic affinity to oxygenated intermediates as a consequence of the electron transfer between single-atom Ir and CoOOH. For Ir_1_/V_O_–CoOOH, the coordinated oxygen of Ir center forms a hydrogen bonding with adjacent *OH/*OOH intermediates, which stabilizes the intermediates and tunes the energy barrier to a theoretically optimal value. This work not only provides insights into selectively anchoring single atoms on specific sites of support surface, but also discloses the correlations between single atoms on different sites and catalytic performance.

## Methods

### Chemicals

Cobalt (II) nitrate hexahydrate (Co(NO_3_)_2_·6H_2_O, ≥98.5%), sodium hydroxide (NaOH, ≥96.0%), sodium hypochlorite (NaOCl, active Cl ≥96.0%), potassium hydroxide (KOH, ≥ 85.0%), active carbon, alcohol (≥99.7%), and Nafion (15–20%) were purchased from Shanghai Chemical Reagent Company. Iridium (IV) chloride hydrate (IrCl_4_·xH_2_O, Ir ≥56.0%) was purchased from Aladdin. All the other chemicals were of analytical grade and used as received without further purification. All aqueous solutions were prepared using deionized water with a resistivity of 18.2 MΩ cm^−1^.

### Synthesis of CoOOH

CoOOH was synthesized using a previously reported method with modifications^19^. Typically, 291 mg of Co(NO_3_)_2_·6H_2_O was dissolved in 200 mL of H_2_O and stirred for 15 min. Then 30 mL of 1 M NaOH solution was added into the above solution and stirred for 30 min. Afterwards, 6 mL of NaOCl was added into the mixture and stirred for 1 h. The precipitate was isolated by centrifugation and washed for four times. Finally, the product was freeze-dried in vacuum freeze-dryer overnight. The as-obtained CoOOH was electrochemically pretreated under oxidative potentials before characterization.

### Synthesis of Ir_1_/T_O_–CoOOH and Ir_1_/V_O_–CoOOH

Ir_1_/T_O_–CoOOH and Ir_1_/V_O_–CoOOH were synthesized via an electrochemical deposition method^23^. The electrochemical deposition was conducted in a standard three-electrode system (CHI 660E, Shanghai CH Instruments), where a glassy carbon electrode (3 mm in diameter), a carbon rod, and an Ag/AgCl electrode was used as the working, counter, and reference electrodes, respectively. The working electrode was prepared by cast 5 μL out of 1.25 mL homogeneous mixture of CoOOH (5 mg), active carbon (5 mg), H_2_O (0.6 mL), alcohol (0.6 mL), and Nafion (50 μL). The yield loading of CoOOH on glassy carbon electrode was 0.28 mg cm^−2^. For the synthesis of Ir_1_/T_O_–CoOOH, the working electrode was first pretreated using a linear sweep method under a potential ranging from 0.10 V to −0.40 V for 10 cycles. Then 100 μM IrCl_4_ was added into the electrolyte as the Ir precursor and the mixture was stirred for 10 min. Then cathodic deposition was carried out using the linear sweep method from 0.10 V to −0.40 V with a sweep rate of 5 mV s^−1^. This deposition was repeated for 10 times. For the synthesis of Ir_1_/V_O_–CoOOH, the working electrode was first pretreated using a linear sweep method under a potential ranging from 1.10 V to 1.80 V for five cycles. Then 100 μM IrCl_4_ was added into the electrolyte as the Ir precursor and the mixture was stirred for 10 min. Then anodic deposition was carried out using the linear sweep method from 1.10 V to 1.80 V with a sweep rate of 5 mV s^−1^. This deposition was repeated for five times. After the deposition, the obtained samples were washed with deionized water and used for later electrochemical measurements. All potentials mentioned in this work were measured against the Ag/AgCl electrode and converted to reversible hydrogen electrode (RHE) scale by *E* (V vs RHE) = *E* (V vs Ag/AgCl) + 0.194 V + 0.0591 pH V. In the given equation, 0.194 V was obtained by calibrating with respect to the reversible hydrogen electrode (RHE). The calibration was carried out in a three-electrode system using a high-purity-hydrogen saturated 0.5 M H_2_SO_4_ electrolyte, where a Pt wire, another Pt wire, and an Ag/AgCl electrode was used as the working, counter, and reference electrodes, respectively. The calibration was conducted using a cyclic voltammetry method with a sweep rate of 1 mV s^−1^. The average of the two potentials at which the current crossed zero was taken to be the thermodynamic potential for the hydrogen electrode reactions.

### XAFS measurements

XAFS spectra at Ir *L*_3_-edge were obtained at the BL14W1 beam line of Shanghai Synchrotron Radiation Facility. The in-situ Ir *L*_3_-edge and Co *K*-edge XAFS spectra were obtained at the 1W1B beam line of Beijing Synchrotron Radiation Facility and BL11B beam line of Shanghai Synchrotron Radiation Facility. The XAFS data were recorded under fluorescence mode. The energy was calibrated according to the absorption edge of pure Ir powder and Co foil. Athena and Artemis codes were used to extract the data and fit the profiles. For the XANES spectra, the experimental absorption coefficients as a function of energies *μ*(*E*) were processed by background subtraction and normalization procedures. We refer to this process as ‘normalized absorption’. For the Ir *L*_3_-edge EXAFS, the Fourier-transformed data in *R* space were analysed by applying the first shell approximation or metallic Ir model for the Ir–O or Ir–Ir shell, respectively. For the in-situ Co *K*-edge EXAFS, the Fourier-transformed data in *R* space were analysed by applying the first shell approximation or metallic Co model for the Co–O or Co–Co shell, respectively. The passive electron factors, *S*_*0*_^*2*^, were determined by fitting the experimental Ir powder data and fixing the Ir–Ir coordination number (*CN*) to be 12. The determined factors were fixed for further analysis of the measured samples. Other parameters such as *CN*, bond distance (*R*), and Debye-Waller (*D. W*.) factor around the absorbed atoms were allowed to vary during the fitting process. XAS spectra at Co *L*-edge were measured at the beam line BL12B of national synchrotron radiation laboratory (NSRL, Hefei).

### Electrochemical measurements

An electrochemical workstation (CHI 660E, Shanghai CH Instruments) was used to evaluate the electrocatalytic reactivities of the samples. The electrochemical measurements were conducted in a standard three-electrode system at room temperature using 1.0 M KOH. The working electrode is the glassy carbon electrode loaded with the catalysts. The counter electrode used a carbon rod. The reference electrode used an Ag/AgCl electrode. The polarization curves towards OER were obtained by linear sweep voltammetry from 1.10 to 1.80 V with a sweep rate of 5 mV s^−1^. The potentials were corrected to compensate for the solution resistance, which were calculated by *E*_*iR*-corrected_ = *E* (V vs RHE) – *iR*, where *i* is the current, and *R* is the uncompensated ohmic electrolyte resistance. *R* is measured to be 9 Ω via high frequency alternating current impedance. The Faradaic efficiency (FE) towards OER was measured in an H-type cell with the cathodic and anodic chambers separated by a membrane. The working electrode and reference electrode were placed in the anodic chamber, while the counter electrode was placed in the cathodic chamber. The Faradaic measurement was conducted at a current density of 10 mA cm^−2^ for 150 min. During the process, argon was continuously sparged into the anodic chamber at a flow rate of 20 sccm. The gaseous product from the anode was measured in real time by in-line gas chromatography. Electrochemically active surface areas (ECSAs) were acquired according to the equation: ECSA = *R*_f_ *S*, where *R*_f_ is the roughness factor and *S* is the geometric area of working electrode. In this work, *S* = 0.07 cm^−2^. *R*_f_ was determined by *R*_f_ = *C*_dl_ / 60 μF cm^−2^ based on the double-layer capacitance (*C*_dl_) of a smooth oxide surface (60 μF cm^−2^). *C*_dl_ was estimated by plotting the ∆*j* (*j*_*a*_ *−* *j*_*c*_) at 0.75 V vs RHE against scan rates of 20, 40, 60, 80, and 100 mV s^−1^, respectively. ∆*j* was acquired by cyclic voltammetry (CV) measurement under 0.7–0.8 V vs RHE. *j*_*a*_ and *j*_*c*_ responds to the highest and the lowest current density values at 0.75 V, respectively. The specific activities were obtained by normalizing the current densities against ECSA. The mass activities were obtained by normalizing the current densities against the mass loading of CoOOH or Ir on working electrode. The TOFs were calculated by TOF = (*j* × *A*) / (4 × *F* × *m*). In the equation, *j* is the current density at a given overpotential, *A* is the geometric surface area of the electrode, *F* is the Faraday constant, and *m* is the moles of Co or Ir metals on the electrode. Tafel slope (*b*) was determined by fitting polarization curves data to the Tafel equation: *η* = *a* + *b* log |*j*|, where *η* is the overpotential for OER, *j* is the current density at the given overpotential. Electrochemical impedance spectroscopy (EIS) measurements were conducted at 1.57 V. The amplitude of the sinusoidal wave was 5 mV. The frequency scan range was 100 kHz–0.05 Hz. Durability test was conducted in galvanostatic mode at 10 mA cm^−2^ in 1.0 M KOH solution at room temperature.

### DFT calculations

The density-functional theory (DFT) calculations were performed with the Vienna Ab-initio Simulation Package (VASP) codes 5.4 (ref. ^39–41^). The projected augmented wave (PAW) pseudo-potentials, the revised Perdew-Burke-Ernzerhof (RPBE) exchange-correlation functional, and a plane wave cutoff of 520 eV have been used in the calculations^42–44^. For a better description of the 3*d* electrons, the Hubbard effective term U_*eff*_ = 3.3 eV for Co was added to the RPBE functional through the rotationally invariant approach^45^. All periodic slab calculations were carried out using a vacuum spacing of at least 15 Å. All calculations kept the bottom layer fixed. A 3 * 3 * 1 Monkhorst–Pack *k*-point mesh was used for all samples in the calculations. Spin-polarized calculations were identified for all surfaces. The convergence of energy and force were set to be 10^−5^ eV and 0.03 eV/Å, respectively. Van der Waals correction was considered by introducing the DFT-D3 method^46,47^.

### Theoretical evaluation of activity

It was assumed that the theoretical overpotentials of CoOOH, Ir_1_/T_O_–CoOOH, and Ir_1_/V_O_–CoOOH followed the conventional OER mechanism^48^. Referring to previous researches, the computational hydrogen electrode model was used to express the chemical potentials of protons and electrons at a given pH and applied potential^48–52^. Under alkaline conditions, the elementary steps during the OER process involve the formation of adsorbed OH, O, and OOH species on the surface (*) according to the following steps:

1 $${{{{{{\rm{OH}}}}}}}^{{{{{{\rm{-}}}}}}}+* \to * {{{{{\rm{OH}}}}}}+{{{{{{\rm{e}}}}}}}^{{-}}$$

2 $$* {{{{{\rm{OH}}}}}}+{{{{{{\rm{OH}}}}}}}^{{-}}\to * {{{{{\rm{O}}}}}}+{{{{{{\rm{H}}}}}}}_{2}{{{{{\rm{O}}}}}}+{{{{{{\rm{e}}}}}}}^{{-}}$$

3 $$* {{{{{\rm{O}}}}}}+{{{{{{\rm{OH}}}}}}}^{{-}}\to * {{{{{\rm{OOH}}}}}}+{{{{{{\rm{e}}}}}}}^{{-}}$$

4 $$* {{{{{\rm{OOH}}}}}}+{{{{{{\rm{OH}}}}}}}^{{-}}\to * +{{{{{{\rm{O}}}}}}}_{2}+{{{{{{\rm{H}}}}}}}_{2}{{{{{\rm{O}}}}}}+{{{{{{\rm{e}}}}}}}^{{-}}$$

Due to the thermodynamic equivalence of the OER process under alkaline and acidic conditions, we modeled the thermochemistry of OER under acidic conditions^51^. Then, steps 1–4 were modified as:

5 $${{{{{{\rm{H}}}}}}}_{2}{{{{{\rm{O}}}}}}+* \to * {{{{{\rm{OH}}}}}}+{{{{{{\rm{H}}}}}}}^{+}+{{{{{{\rm{e}}}}}}}^{{{{{{\rm{-}}}}}}}-$$

6 $$* {{{{{\rm{OH}}}}}}+{{{{{{\rm{H}}}}}}}_{2}{{{{{\rm{O}}}}}}\to * {{{{{\rm{O}}}}}}+{{{{{{\rm{H}}}}}}}_{2}{{{{{\rm{O}}}}}}+{{{{{{\rm{H}}}}}}}^{+}+{{{{{{\rm{e}}}}}}}^{{{{{{\rm{-}}}}}}}$$

7 $$* {{{{{\rm{O}}}}}}+{{{{{{\rm{H}}}}}}}_{2}{{{{{\rm{O}}}}}}\to * {{{{{\rm{OOH}}}}}}+{{{{{{\rm{H}}}}}}}^{+}+{{{{{{\rm{e}}}}}}}^{{-}}$$

8 $$* {{{{{\rm{OOH}}}}}}+{{{{{{\rm{H}}}}}}}_{2}{{{{{\rm{O}}}}}}\to * +{{{{{{\rm{O}}}}}}}_{2}+{{{{{{\rm{H}}}}}}}_{2}{{{{{\rm{O}}}}}}+{{{{{{\rm{H}}}}}}}^{+}+{{{{{{\rm{e}}}}}}}^{{{{{{\rm{-}}}}}}}$$

Thus, the Gibbs free energy change for steps 5–8 can be expressed as:

9 $$\triangle {G}_{1}={\triangle G}_{* {{{{{\rm{OH}}}}}}}-{eU}+{\triangle G}_{{{{{{\rm{H}}}}}}+}({{{{{\rm{pH}}}}}})$$

10 $$\triangle {G}_{2}={\triangle G}_{* {{{{{\rm{O}}}}}}}-{\triangle G}_{* {{{{{\rm{OH}}}}}}}-{eU}+{\triangle G}_{{{{{{\rm{H}}}}}}+}\left({{{{{\rm{pH}}}}}}\right)$$

11 $${\triangle G}_{3}={\triangle G}_{* {{{{{\rm{OOH}}}}}}}-{\triangle G}_{* {{{{{\rm{O}}}}}}}-{eU}+{\triangle G}_{{{{{{\rm{H}}}}}}+}({{{{{\rm{pH}}}}}})$$

12 $${\triangle G}_{4}=4.92\,[{eV}]-{\triangle G}_{* {{{{{\rm{OOH}}}}}}}-{eU}+{\triangle G}_{{{{{{\rm{H}}}}}}+}({pH})$$

where *U* represents the potential measured against normal hydrogen electrode (NHE) at standard conditions (*T* = 298.15 K, *P* = 1 bar, pH = 0); ∆*G*_H+_(pH) = −k_B_Tln(10) × pH is the free energy change at a nonzero pH value. Because the O_2_ bond energy is difficult to determine by DFT calculations, the sum of −∆*G*_1*-*4_ was fixed to the experimental Gibbs free energy of −4.92 eV for forming two O_2_ molecules. The Gibbs free energy corrections of *OH, *O, and *OOH intermediates include zero-point energy (ZPE) and entropy corrections according to ∆*G* = ∆*E* + ZPE - T∆*S*. The influence of aqueous environment was considered by correcting the calculated T∆*S* value of −0.58 eV with an experimental T∆*S* value of −0.67 eV under 0.035 bar at 298.15 K^52^. The sum of all corrections on *OH, *O, and *OOH were set as 0.35, 0.05, and 0.40, respectively, using the harmonic approximation^38,50^. The theoretical overpotential was then defined as:

13 $$\eta ={\max }({\triangle} {G}_{1},\,{\triangle} {G}_{2},\,{\triangle} {G}_{3},\,{\triangle} {G}_{4})/e-1.23\,[{{{{{\mathrm{V}}}}}}]$$

### Instrumentations

TEM images were taken on Hitachi H-7650 transmission electron microscope operating at an acceleration voltage of 100 kV. XRD patterns were recorded using a Philips X’Pert Pro Super diffractometer with Cu-Kα radiation (λ = 1.54178 Å). All HAADF-STEM images and EDX elemental mapping without further statement were carried out on JEOL ARM-200F field-emission transmission electron microscope operating at an accelerating voltage of 200 kV using Cu-based TEM grids. The HAADF-STEM images in Supplementary Fig. 4 were carried out on Nion HERMES-100 under an accelerating voltage of 60 kV. ESR spectrum was measured on an ESR spectrometer (JEOL JES-FA200) at 300 K and 9.062 GHz. XPS measurements were performed on a VG ESCALAB MK II X-ray photoelectron spectrometer with Mg Kα = 1253.6 eV as the exciting source. ICP-AES (Atomscan Advantage, Thermo Jarrell Ash, USA) analyses were used to determine the mass loadings of metal species. Raman spectrum were collected using a laser Raman analyzer LabRAM HR (Horiba/Jobin Yvon, Longjumeau) equipped with a frequency doubled Nd:YAG 532.1 nm laser. The gas products of OER were monitored by an online GC (SHIMADZU, GC-2014) equipped with a thermal conductivity detector and Molsieve 5 A colum.

## Supplementary information

Supplementary Information

## Data Availability

All data is available in the main text or the supplementary information. Source data are provided with this paper.

## Source data

Source Data
