# Supplementary material for: Selectively anchoring single atoms on specific sites of supports for improved oxygen evolution
Source: Nat Commun. 2022 May 5;13:2473. doi: 10.1038/s41467-022-30148-3 (PMC9072319; doi:10.1038/s41467-022-30148-3)
Supplement: Supplementary file 1 — Supplementary Information [file 41467_2022_30148_MOESM1_ESM.pdf]

Supplementary Information for

Selectively anchoring single atoms on specific sites of supports for improved oxygen evolution

Zhirong Zhang[1,2†], Chen Feng[1†], Dongdi Wang[2†], Shiming Zhou[1]*, Ruyang Wang[2], Sunpei Hu[1], Hongliang Li[1], Ming Zuo[1], Yuan Kong[1]*, Jun Bao[2]*, Jie Zeng[1,3]*

[1]Hefei National Research Center for Physical Sciences at the Microscale, Key Laboratory of Strongly-Coupled Quantum Matter Physics of Chinese Academy of Sciences, University of Science and Technology of China, Hefei, Anhui 230026, P. R. China

[2]National Synchrotron Radiation Laboratory, University of Science and Technology of China, Hefei, Anhui 230026, P. R. China

[3]Key Laboratory of Surface and Interface Chemistry and Energy Catalysis of Anhui Higher Education Institutes, Department of Chemical Physics, University of Science and Technology of China, Hefei, Anhui 230026, P. R. China

*Corresponding author. E-mail: zhousm@ustc.edu.cn (S.Z.); kongyuan@ustc.edu.cn (Y.K.); baoj@ustc.edu.cn (J.B.); zengj@ustc.edu.cn (J.Z.)

[†]These authors contributed equally to this work.

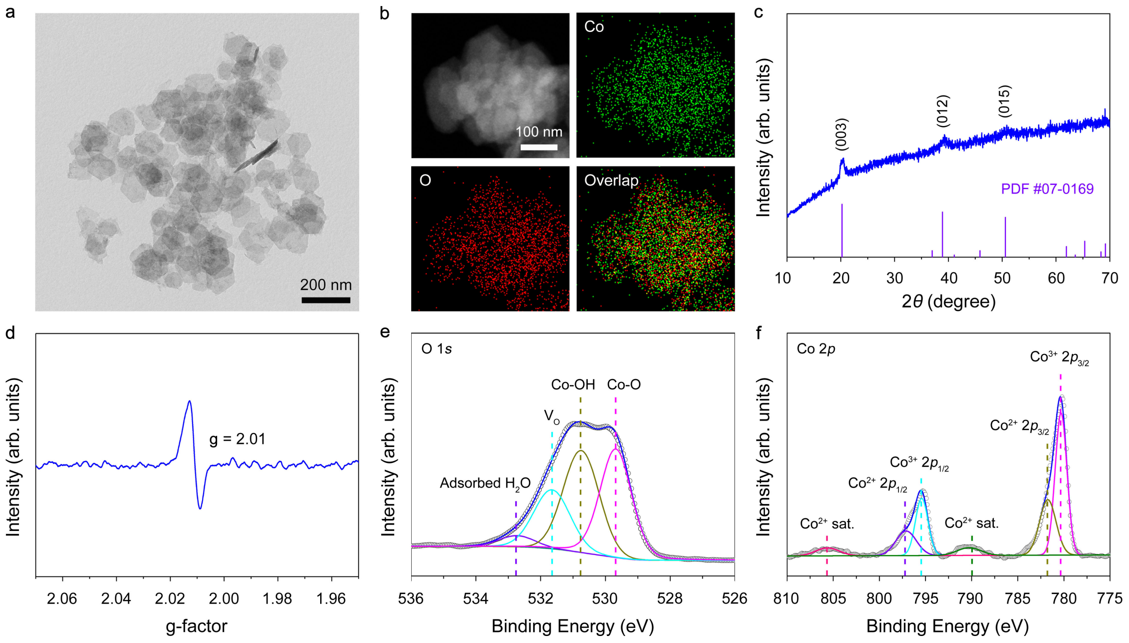

Supplementary Fig. 1 | Morphological and structural characterizations of CoOOH nanosheets. a, TEM image. **b**, EDX elemental mapping. **c**, XRD pattern. **d**, ESR spectrum. **e**, O 1*s* XPS spectrum. **f**, Co 2*p* XPS spectrum.

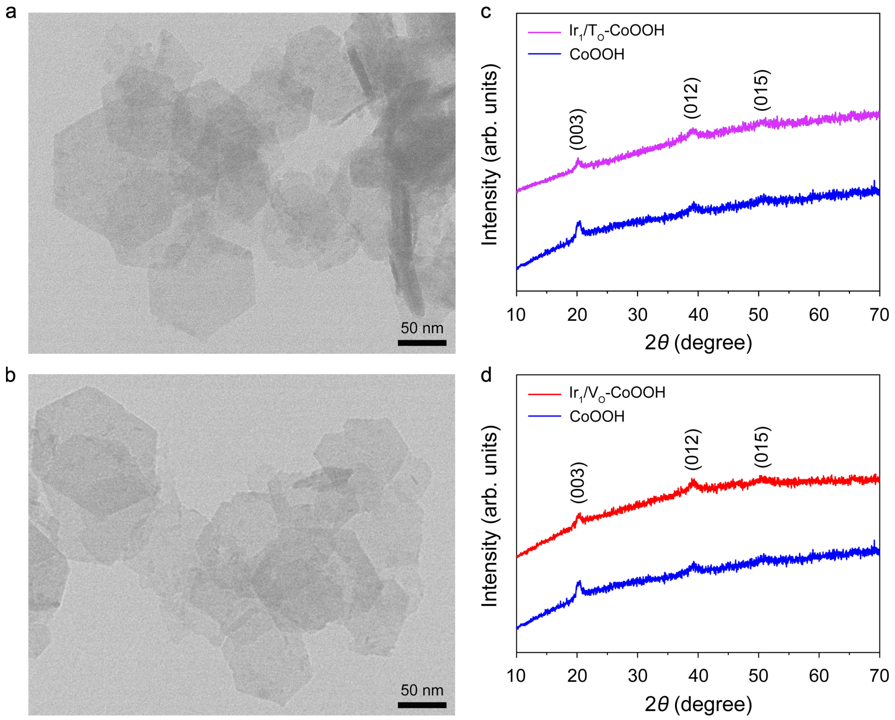

Supplementary Fig. 2 | Morphological and structural characterizations of Ir_1/T_O-CoOOH and Ir_1/V_O-CoOOH. a, **b**, TEM images of Ir_1/T_O-CoOOH (**a**) and Ir_1/V_O-CoOOH (**b**). **c**, **d**, XRD patterns of Ir_1/T_O-CoOOH (**c**) and Ir_1/V_O-CoOOH (**d**).

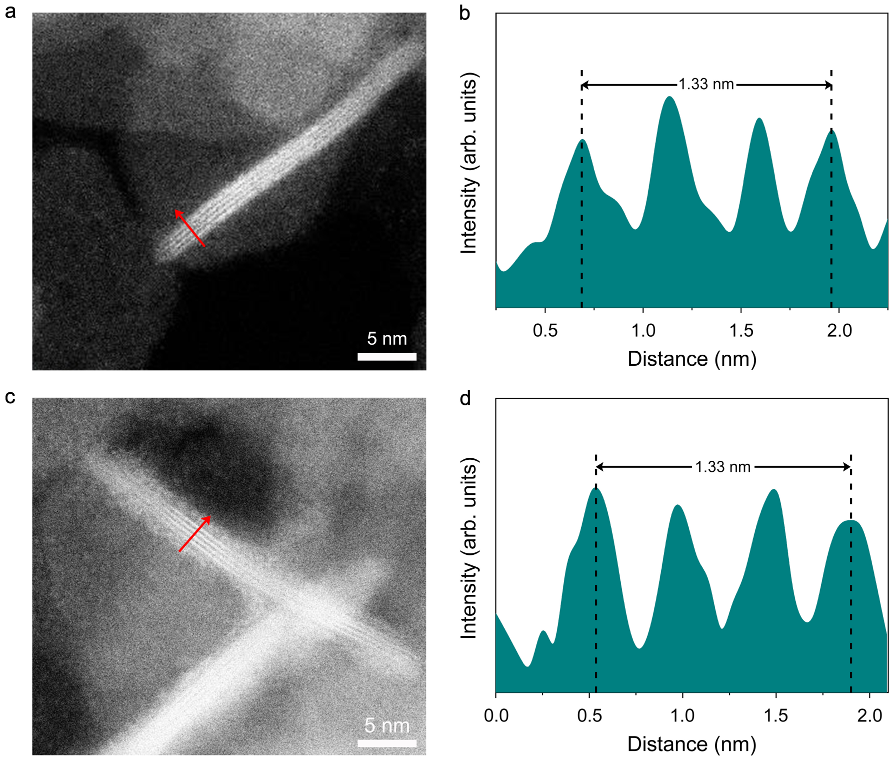

Supplementary Fig. 3 | Structural characterizations of Ir_1/T_O-CoOOH and Ir_1/V_O-CoOOH from the side view of CoOOH nanosheets. a, HAADF-STEM image of Ir_1/T_O-CoOOH. **b**, Line intensity profile obtained from the indicated direction in the HAADF-STEM image of Ir_1/T_O-CoOOH. **c**, HAADF-STEM image of Ir_1/V_O-CoOOH. **d**, Line intensity profile obtained from the indicated direction in the HAADF-STEM image of Ir_1/V_O-CoOOH.

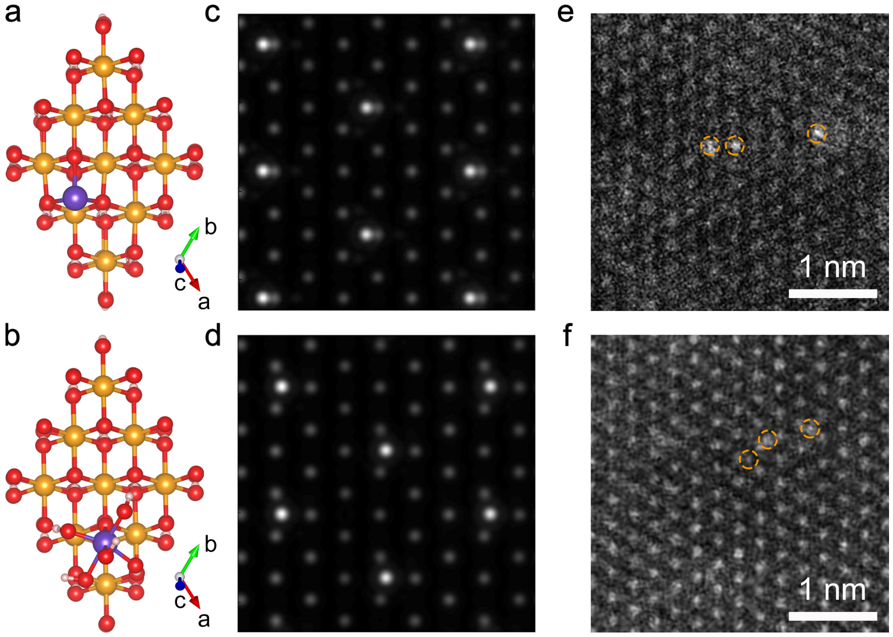

Supplementary Fig. 4 | Imaging of the anchoring sites of Ir single atoms on CoOOH from [-111] projection. a-b, Schematic structure models of Ir single atoms on three-fold hollow site (**a**) and oxygen vacancy site (**b**) of CoOOH from [-111] projection. The pink, red, orange, and purple spheres represent H, O, Co, and Ir atoms, respectively. **c-d**, Simulated HAADF-STEM images of Ir single atoms on three-fold hollow site (**c**) and oxygen vacancy site (**d**) of CoOOH from [-111] projection. **e-f**, HAADF-STEM images of Ir_1/T_O-CoOOH (**c**) and Ir_1/V_O-CoOOH (**d**) from [-111] projection. Ir single atoms are indicated by yellow circles. In the simulated HAADF-STEM images from [-111], Ir atoms at three-fold hollow sites almost overlap with the Co column, while Ir atoms at oxygen vacancy sites are located in the interstice of three triangular Co columns. Therefore, we can resolve the precise anchoring sites of Ir single atoms according to the HAADF-STEM images from [-111] projection. Of note, despite Ir has larger Z-contrast than Co, the Ir single atoms showed less brightness than the Co lattice sites in the HAADF-STEM image of Ir_1/Vo-CoOOH from [-111] projection. This is because the brightness of Co lattice sites is the sum of several Co atoms in a projection column, while the brightness of Ir comes from a single atom.

Supplementary Table 1 | EXAFS fitting results of Ir_1/T_O-CoOOH and Ir_1/V_O-CoOOH. Ir powder and IrO_2 were used as references.

Samples	Ir-O		Ir-Cl		Ir-Ir		*D. W.*	ΔE_0 (eV)
	R (Å)	*CN*	*R* (Å)	*CN*	*R* (Å)	*CN*		
Ir_1/T_O-CoOOH	1.97±0.04	3.3±0.7	2.32±0.04	3.2±0.8	—	—	0.002 (O)	8.8±2.1
Ir_1/V_O-CoOOH	2.01±0.01	5.9±0.5	—	—	—	—	0.006 (Cl)	16.0±1.0
Ir powder	—	—	—	—	2.71	12.0	0.004	7.9±1.3
IrO_2	1.98±0.01	6.0	—	—	—	—	0.002±0.001	15.8±2.2

R, distance between absorber and backscatter atoms; *CN*, coordination number; *D. W.*, Debye-Waller factor; ΔE_0, inner potential correction that accounts for the difference in the inner potential between the sample and the references.

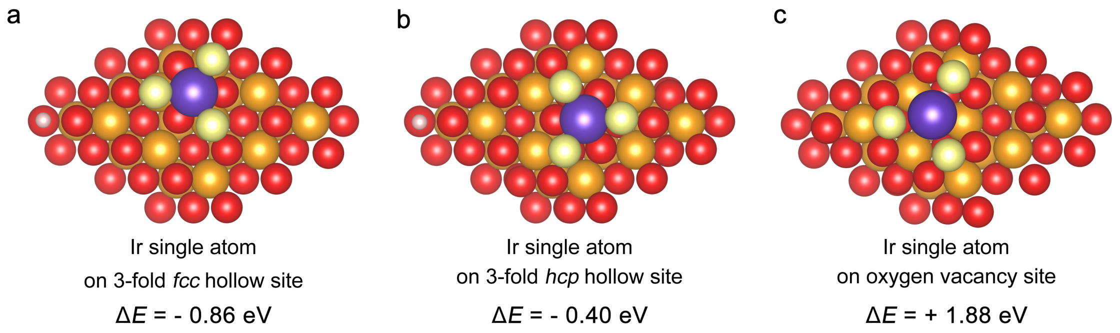

Supplementary Fig. 5 | Formation energies (Δ*E*) of $IrCl_3^+$ anchoring on three-fold *fcc* (a), three-fold *hcp* hollow site (b), and oxygen vacancy site (c) of CoOOH (001). The pink, red, yellow, orange, and purple spheres represent H, O, Cl, Co, and Ir atoms, respectively. The formation energy (Δ*E*) was calculated by DFT.

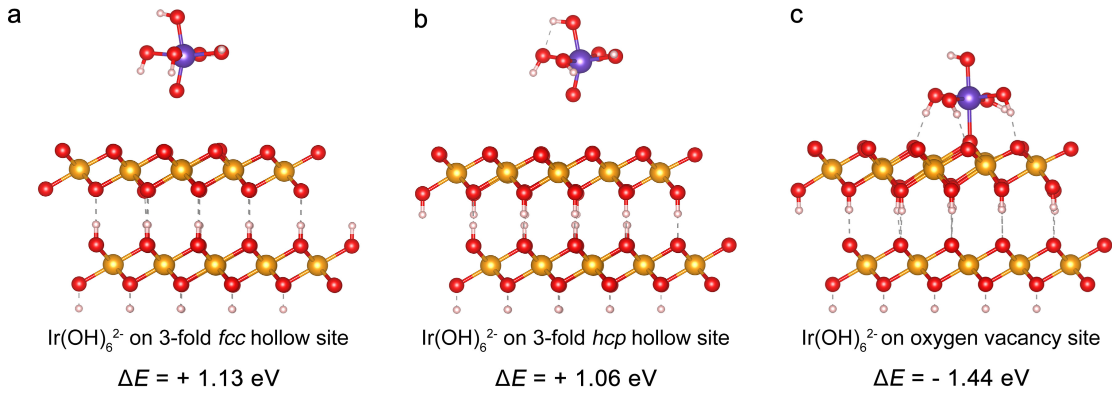

Supplementary Fig. 6 | Formation energies (ΔE) of $Ir(OH)_6^{2-}$ anchoring on three-fold *fcc* (a), three-fold *hcp* hollow site (b), and oxygen vacancy site (c) of CoOOH (001). The pink, red, orange, and purple spheres represent H, O, Co, and Ir atoms, respectively. The formation energy (ΔE) was calculated by DFT.

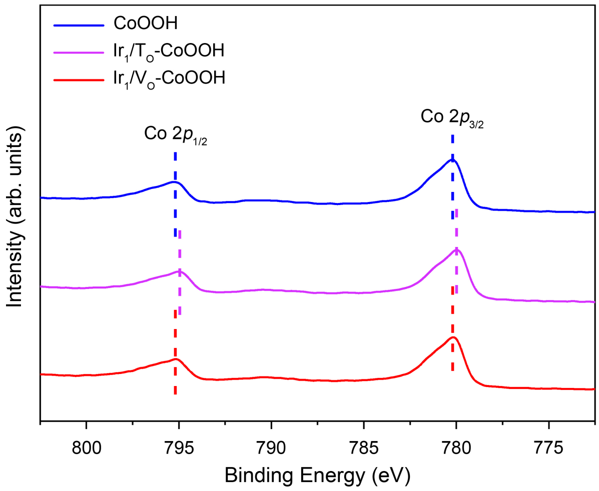

Supplementary Fig. 7 | Electronic structure characterization. Co 2*p* XPS spectra of CoOOH, Ir_1/T_O-CoOOH, and Ir_1/V_O-CoOOH.

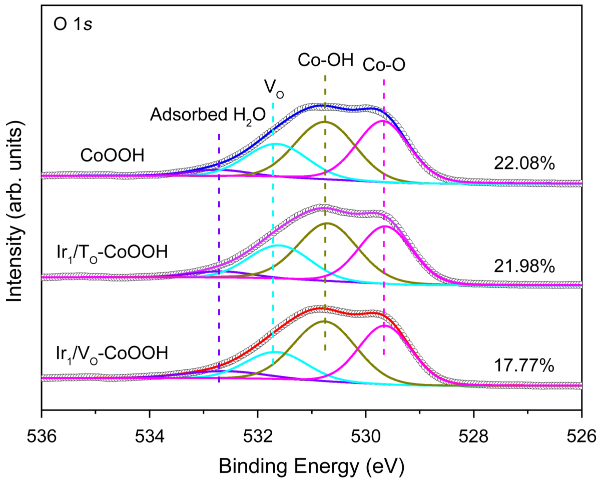

Supplementary Fig. 8 | O 1*s* XPS spectra of Ir$_1$/T$_O$-CoOOH and Ir$_1$/V$_O$-CoOOH after anchoring Ir single atoms. The O 1*s* XPS spectrum of CoOOH was used as a reference.

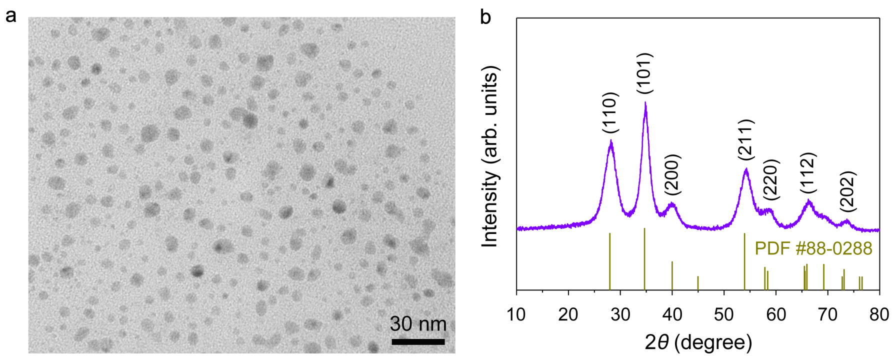

Supplementary Fig. 9 | Morphological and structural characterizations of commercial IrO_2. **a**, TEM image. **b**, XRD pattern.

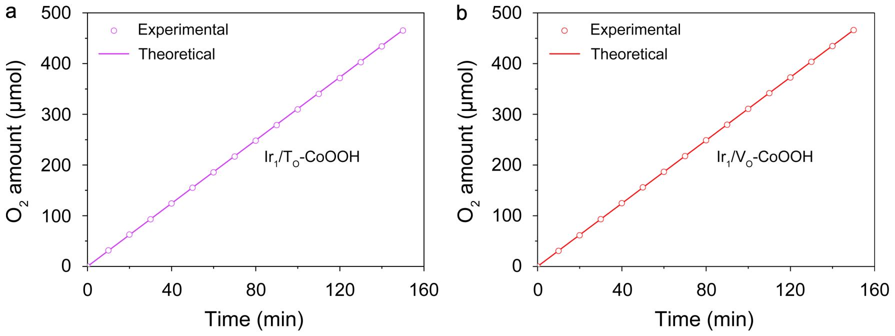

Supplementary Fig. 10 | Faradaic efficiency of Ir_1/T_O-CoOOH (a) and Ir_1/V_O-CoOOH (b) for OER. The measurement was conducted at a current density of 10 mA cm^{-2} for 150 min. The experimental amount of O_2 production matches well with the theoretical amount, indicating over 99% Faradaic efficiency for OER.

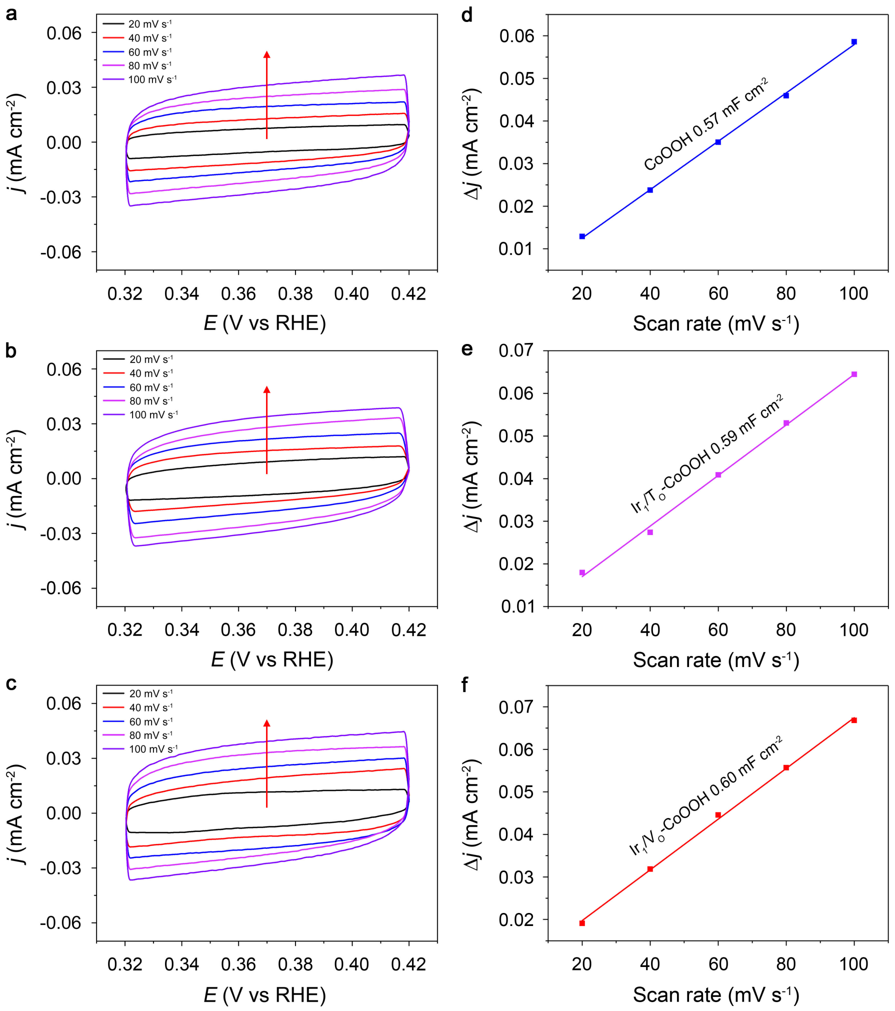

Supplementary Fig. 11 | CV curves and charging current density differences of CoOOH, Ir_1/T_O-CoOOH, and Ir_1/V_O-CoOOH. a-c, CV curves of CoOOH (**a**), Ir_1/T_O-CoOOH (**b**), and Ir_1/V_O-CoOOH (**c**), respectively. **d-f**, Charging current density differences of CoOOH (**d**), Ir_1/T_O-CoOOH (**e**), and Ir_1/V_O-CoOOH (**f**), respectively.

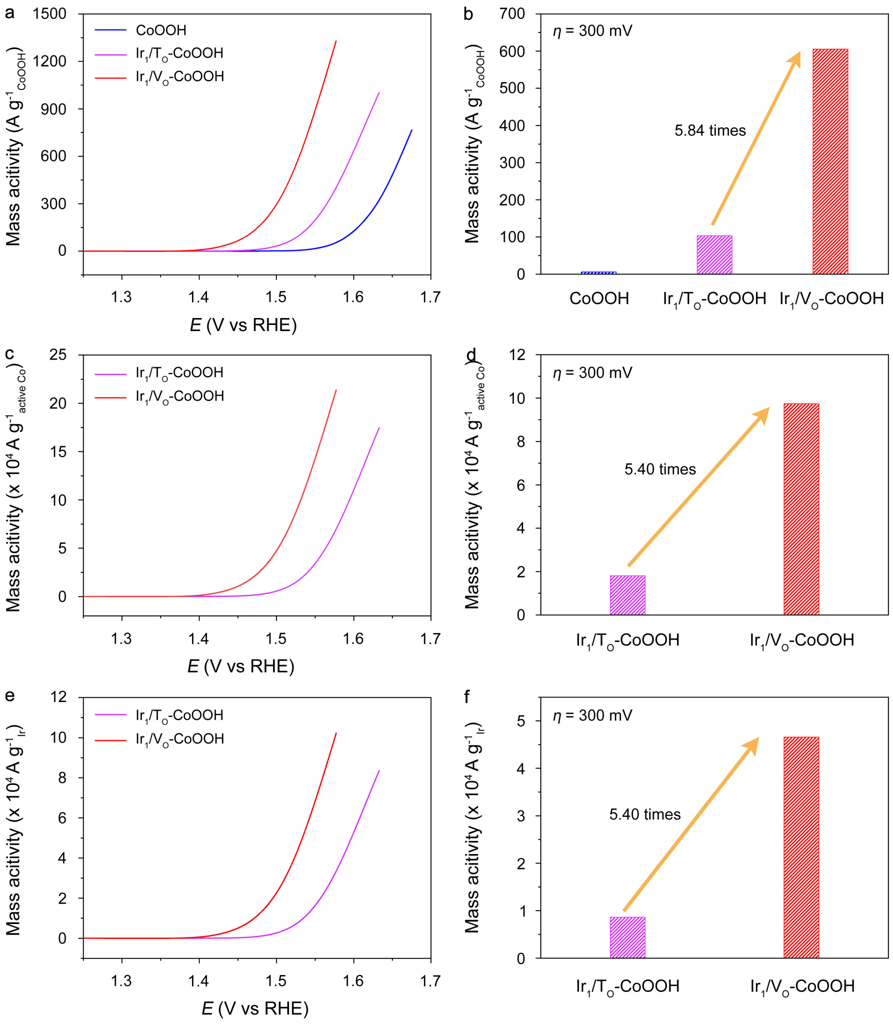

Supplementary Fig. 12 | Mass activities of CoOOH, Ir1/TO-CoOOH, and Ir1/VO-CoOOH normalizing to different components. a, Polarization curves normalizing to the overall mass of CoOOH. **b**, Mass activities normalizing to the overall mass of CoOOH at an overpotential of 300 mV. **c**, Polarization curves normalizing to the mass of active CoOOH. CoOOH was activated by adjacent Ir single atoms, so that the number of active Co sites could be roughly estimated using the number of Ir atoms. **d**, Mass activities normalizing to the mass of active CoOOH at an overpotential of 300 mV. **e**, Polarization curves normalizing to the mass of Ir single atoms. **f**, Mass activities normalizing to the mass of Ir single atoms at an overpotential of 300 mV.

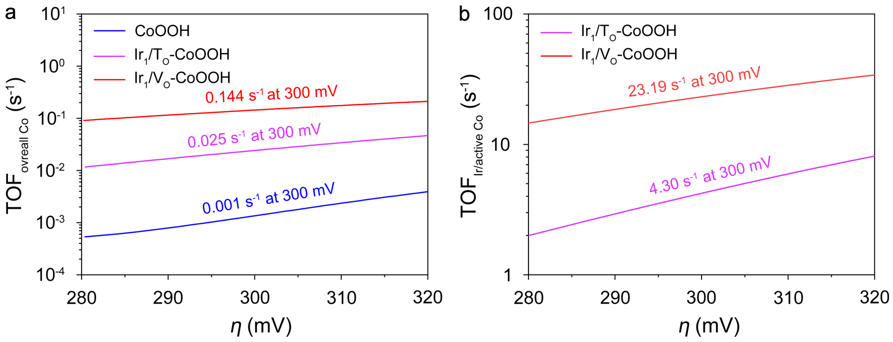

Supplementary Fig. 13 | TOFs normalizing to different components at different overpotentials (*η*). **a**, TOFs of CoOOH, Ir_1/T_O-CoOOH, and Ir_1/V_O-CoOOH normalizing to the number of Co sites. **b**, TOFs of Ir_1/T_O-CoOOH and Ir_1/V_O-CoOOH normalizing to the number of Ir or active Co sites.

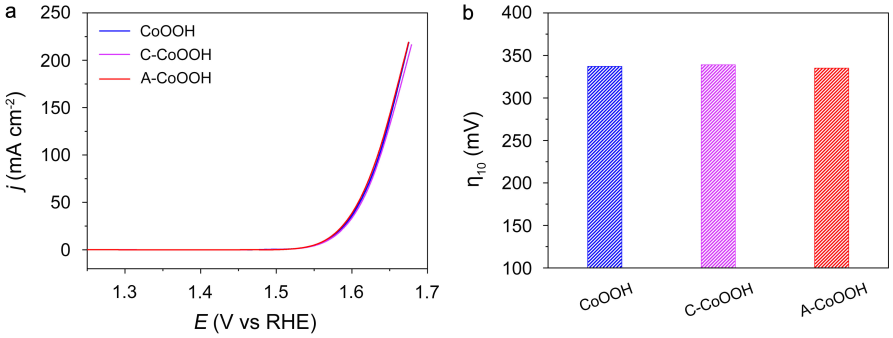

Supplementary Fig. 14 | OER performance of CoOOH treated by electrochemical methods without anchoring Ir single atoms. a, Polarization curves of CoOOH, C-CoOOH, and A-CoOOH. **b**, Overpotentials at 10 mA cm^{-2} for CoOOH, C-CoOOH, and A-CoOOH. C-CoOOH was CoOOH treated with the same cathodic electrochemical method as in synthesizing Ir_1/T_O-CoOOH without adding $IrCl_4$. A-CoOOH was CoOOH treated with the same anodic electrochemical method as in synthesizing Ir_1/V_O-CoOOH without adding $IrCl_4$.

Supplementary Table 2 | Comparison of oxygen evolution performance for reported Co-based catalysts in alkaline electrolyte.

Catalysts	Electrolyte	Overpotential (mV) @ j = 10 mA cm^{-2}	Tafel Slope (mV dec^{-1})	Ref.
Ir_1/V_O-CoOOH	1 M KOH	200	32	This work
Ir_1/T_O-CoOOH		270	65	
Ru/CoFe-LDHs	1 M KOH	198	39	1
Fe-CoP/CoO	1 M KOH	219	52	2
MoCo1.5C-700	1 M KOH	232.5	61	3
CoIr-0.2	1 M KOH	235	70.2	4
$Zn_{0.2}Co_{0.8}OOH$	1 M KOH	235	34.7	5
NiCo-UMOFNs	1 M KOH	250	42	6
o-$CoSe_2$-O ultrathin nanosheets	1 M KOH	251	73	7
a-$CoVO_x$	1 M KOH	254	34	8
NiCo LDH-TPA	1 M KOH	267	52.4	9
CoP@RGO	1 M KOH	280	75	10
30 % Pt/$LiCoO_2$	1 M KOH	285	46.8	11
Cu-$Co(OH)_2$	1 M KOH	300	47	12
Pt-CoS_2/CC	1 M KOH	300	49	13
CoO_x + Fe^{3+}	1 M KOH	309	27.6	14
Co/CNFs	1 M KOH	320	79	15
NiCo LDHs	1 M KOH	334	41	16

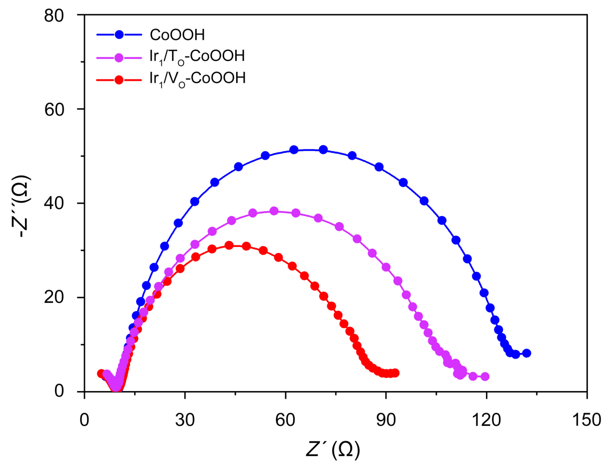

Supplementary Fig. 15 | Electocatalytic characterization. Electrochemical impedance spectra of CoOOH, Ir_1/T_O-CoOOH, and Ir_1/V_O-CoOOH.

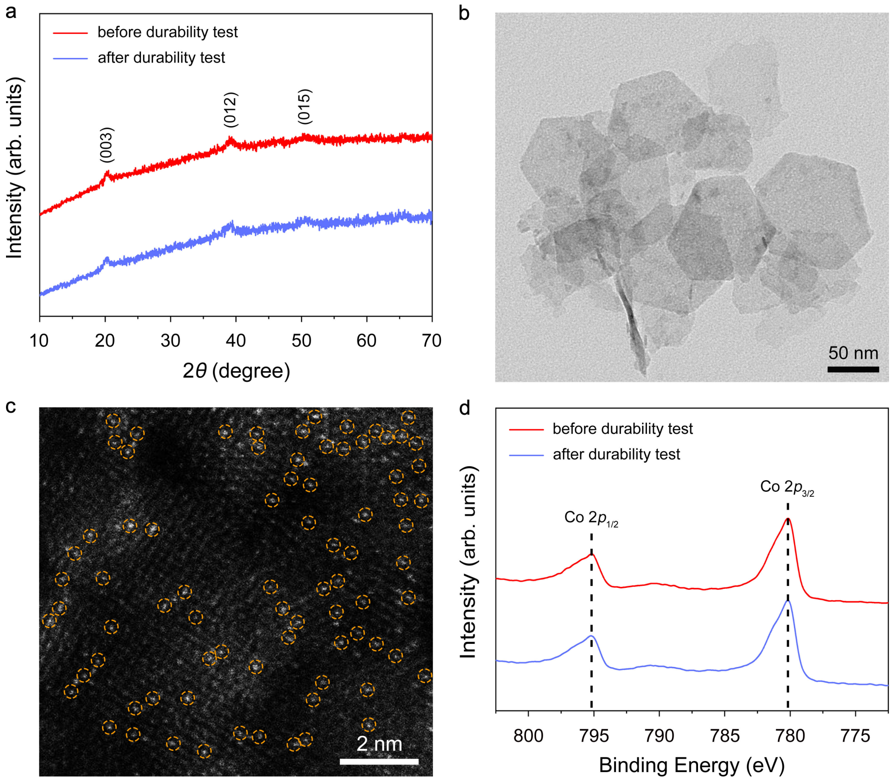

Supplementary Fig. 16 | Morphological and structural characterizations of Ir_1/V_O-CoOOH after durability test. a, XRD pattern. **b**, TEM image. **c**, HAADF-STEM image. **d**, Co 2*p* XPS spectrum. XRD pattern and 2*p* XPS spectrum of Ir_1/V_O-CoOOH before durability test were used as references.

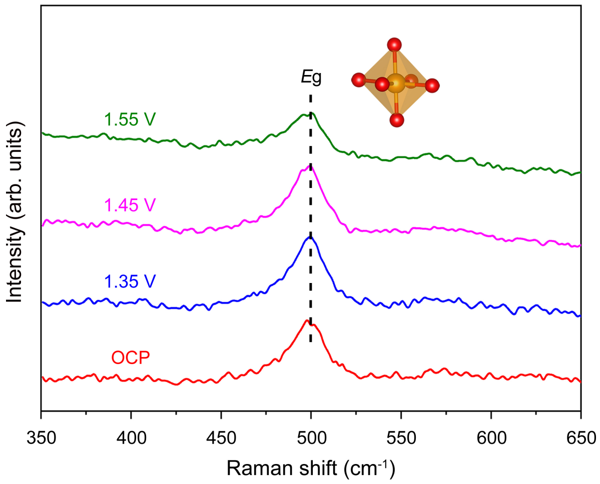

Supplementary Fig. 17 | Structural characterization. *In-situ* Raman spectra of Ir_1/V_O-CoOOH at different applied potentials.

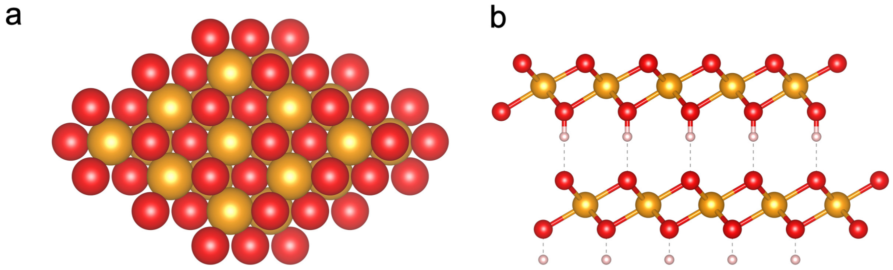

Supplementary Fig. 18 | Schematic structure model of CoOOH. **a**, Top view. **b**, Side view. The pink, red, and orange spheres represent H, O, and Co atoms, respectively.

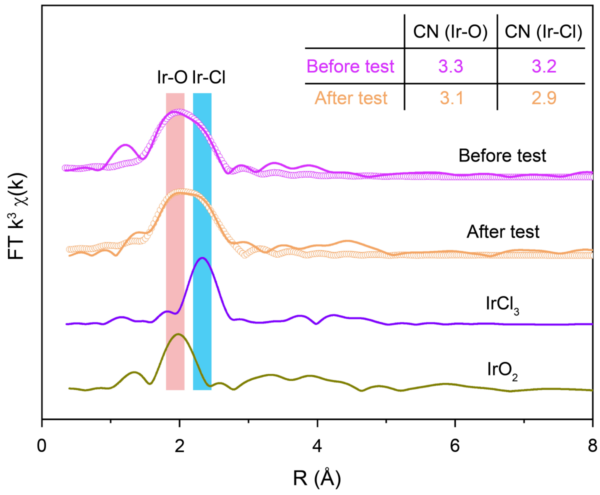

Supplementary Fig. 19 | Normalized Ir L_3-edge EXAFS spectra of Ir_1/T$_O$-CoOOH before and after OER measurements. The experimental and fitting results are shown in solid lines and circles, respectively.

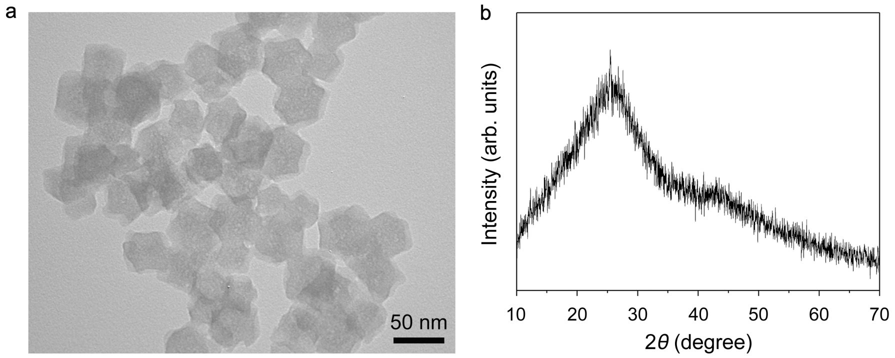

Supplementary Fig. 20 | Morphological and structural characterizations of nitrogen-doped carbon (N-C). a, TEM image. **b**, XRD pattern.

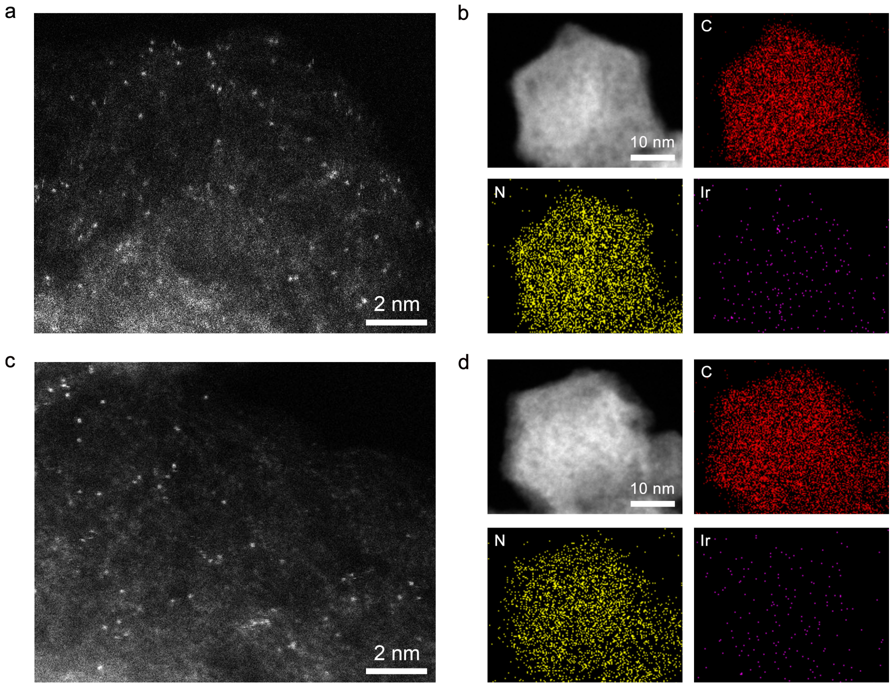

Supplementary Fig. 21 | Structural characterizations of Ir single atoms on nitrogen-doped carbon (N-C). a, **b**, HAADF-STEM image (**a**) and EDX elemental mapping (**b**) of C-Ir_1/N-C. **c**, **d**, HAADF-STEM image (**c**) and EDX elemental mapping (**d**) of A-Ir_1/N-C. Singly-dispersed Ir atoms are indicated by yellow circles.

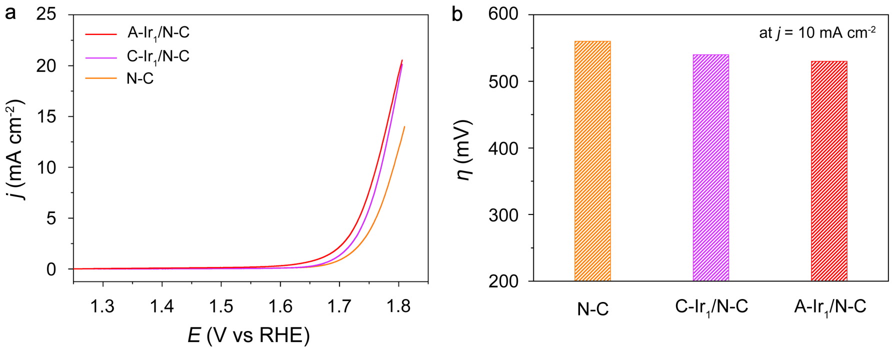

Supplementary Fig. 22 | Electrocatalytic performance of N-C, C-Ir1/N-C, and A-Ir1/N-C towards OER. a, Polarization curves. **b**, Overpotentials at 10 mA cm^{-2}.

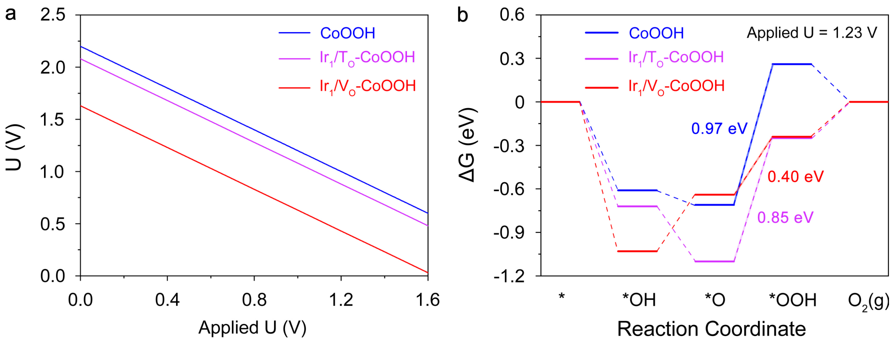

Supplementary Fig. 23 | Correlations between applied potential (U) and OER performance. **a**, Equilibrium potential as a function of applied potentials (U) for CoOOH, Ir_1/T_O-CoOOH, and Ir_1/V_O-CoOOH. **b**, Free energy diagram of CoOOH, Ir_1/T_O-CoOOH, and Ir_1/V_O-CoOOH at an applied potential of 1.23 V.

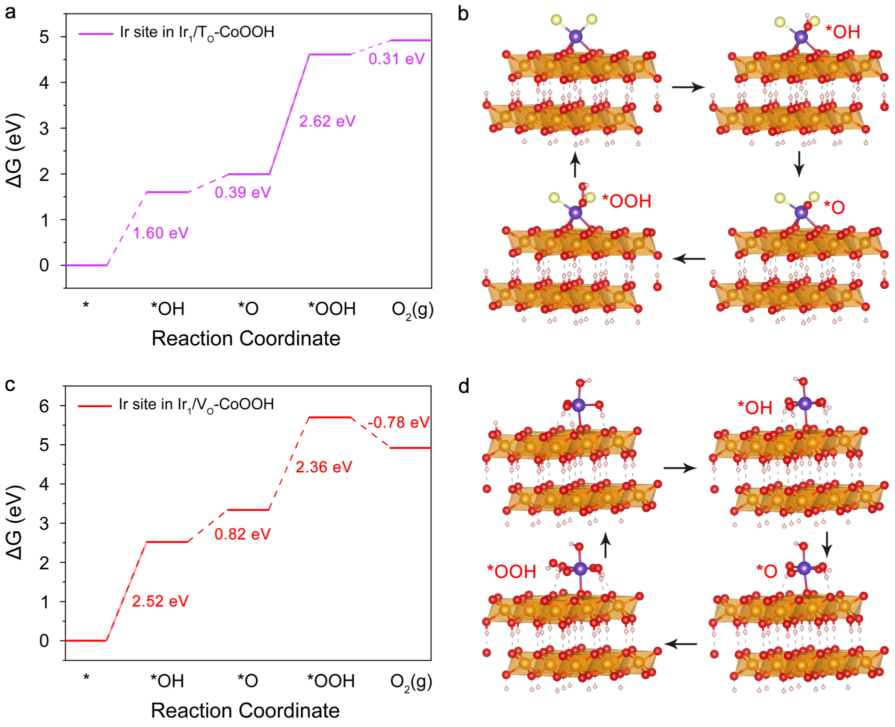

Supplementary Fig. 24 | OER mechanism on single Ir site. a-b, Free energy diagram (**a**) and schematic OER pathway (**b**) of Ir_1/T_O-CoOOH. **c-d**, Free energy diagram (**c**) and schematic OER pathway (**d**) of Ir_1/V_O-CoOOH. The pink, red, yellow, orange, and purple spheres represent H, O, Cl, Co, and Ir atoms, respectively. The dashed grey line indicates hydrogen bonding.

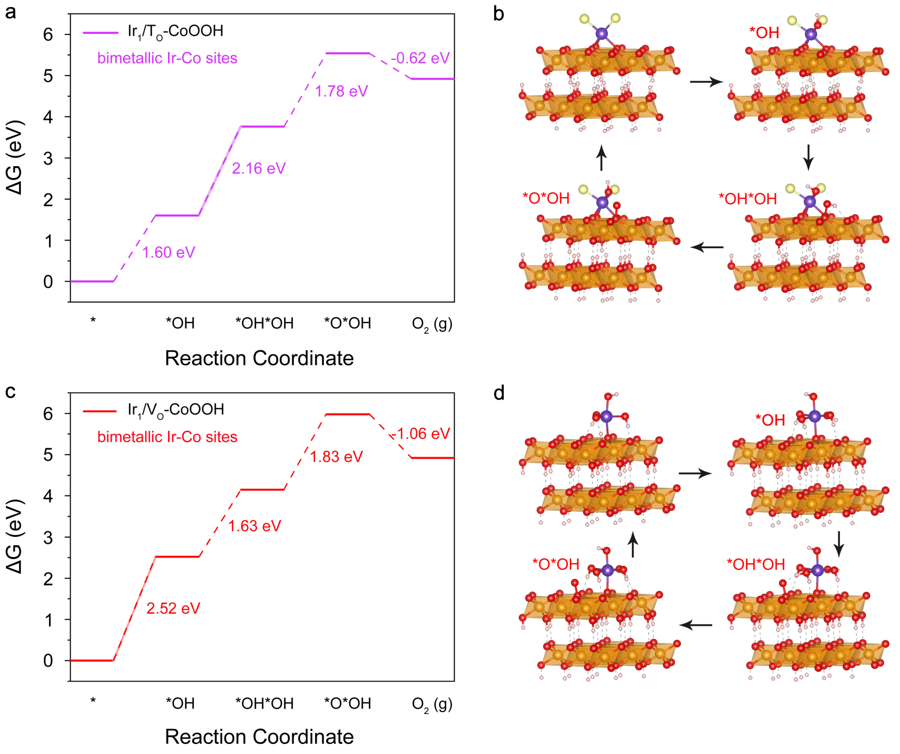

Supplementary Fig. 25 | OER mechanism on bimetallic Ir-Co sites. a-b, Free energy diagram (**a**) and schematic OER pathway (**b**) of Ir_1/T_O-CoOOH. **c-d**, Free energy diagram (**c**) and schematic OER pathway (**d**) of Ir_1/V_O-CoOOH. The pink, red, yellow, orange, and purple spheres represent H, O, Cl, Co, and Ir atoms, respectively. The dashed grey line indicates hydrogen bonding. The calculations were based on a dual-site mechanism, during which *OH adsorbed on two adjacent Ir and Co sites directly coupled to form oxygen. The elementary steps are as follows[17]:

$OH^- + * \rightarrow *OH + e^-$ (1)

$*OH + OH^- \rightarrow *OHOH + e^-$ (2)

$*OHOH + OH^- \rightarrow *OH{-}{-}{-}O + H_2O + e^-$ (3)

$*OH{-}{-}{-}O + OH^- \rightarrow O_2 + H_2O + e^-$ (4)

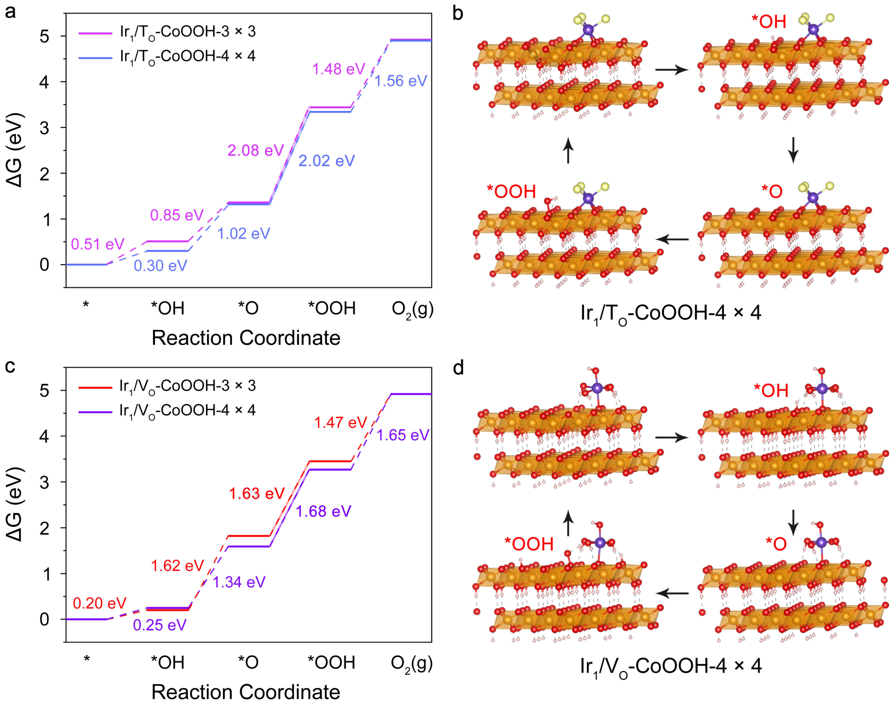

Supplementary Fig. 26 | OER mechanism using a two-layered 4*4 supercell of CoOOH for calculation. a-b, Free energy diagram (**a**) and schematic OER pathway (**b**) of Ir_1/T_O-CoOOH. **c-d**, Free energy diagram (**c**) and schematic OER pathway (**d**) of Ir_1/V_O-CoOOH. The pink, red, yellow, orange, and purple spheres represent H, O, Cl, Co, and Ir atoms, respectively. The dashed grey line indicates hydrogen bonding.

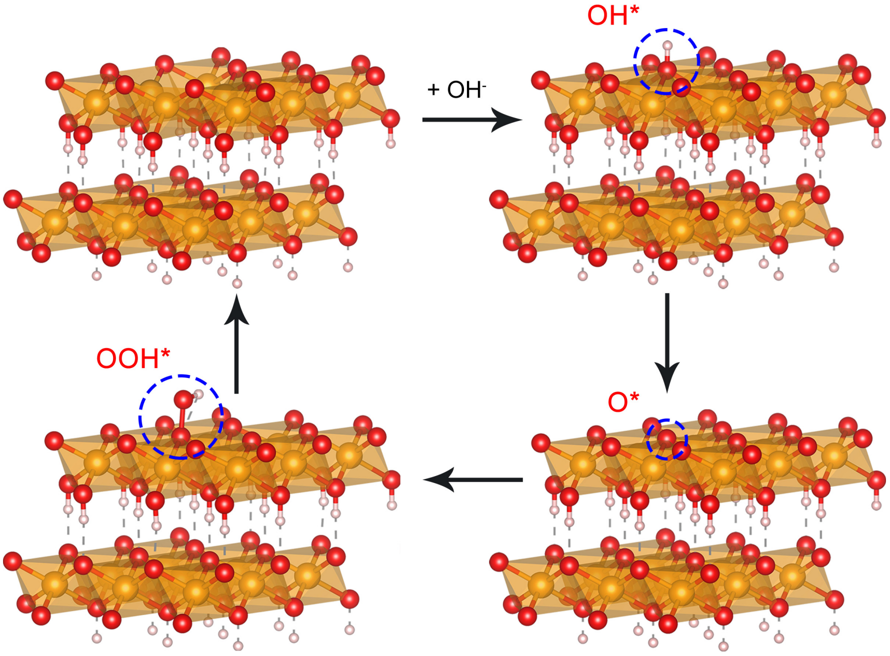

Supplementary Fig. 27 | Reaction pathway of CoOOH towards OER. A dashed grey line indicates the hydrogen bonding between the CoOOH layers. The pink, red, and orange balls represent H, O, and Co atoms, respectively. The reaction intermediates are indicated by blue circles. The reaction started from the adsorption of OH^- ion, as followed by the sequential deprotonation to form O*, O-O bonding formation to generate OOH*, and desorption to produce oxygen.

Supplementary Table 3 | Gibbs free energies (Δ*G*) of OER intermediates *OH, *O, and *OOH on CoOOH, Ir_1/T_O-CoOOH, and Ir_1/V_O-CoOOH.

Samples	ΔG_{*OH} (eV)	ΔG_{*O} (eV)	ΔG_{*OOH} (eV)
CoOOH	0.62	1.75	3.95
Ir_1/T_O-CoOOH	0.51	1.36	3.44
Ir_1/V_O-CoOOH	0.20	1.82	3.45

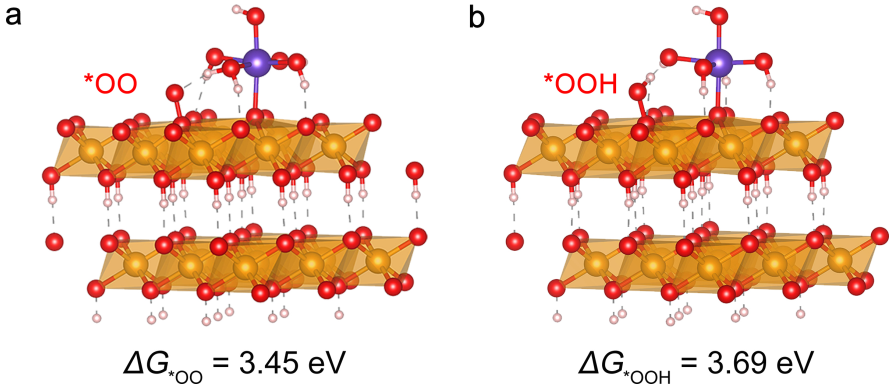

Supplementary Fig. 28 | Gibbs free energies (ΔG_{*OOH}) of *OOH on Ir_1/V_O-CoOOH with and without deprotonation. **a**, *OO with deprotonation. The hydrogen of *OOH was deprotonated to adsorb on the atomic Ir species in the presence of hydrogen bonding. **b**, *OOH without deprotonation. The hydrogen of *OOH was not deprived by the hydrogen bonding.

Supplementary References

1. Li, P. et al. Boosting oxygen evolution of single-atomic ruthenium through electronic coupling with cobalt-iron layered double hydroxides. *Nat. Commun.* **10**, 1711 (2019).
2. Hu, X. et al. 2D Fe-containing cobalt phosphide/cobalt oxide lateral heterostructure with enhanced activity for oxygen evolution reaction. *Nano Energy* **56**, 109-117 (2019).
3. Shah, M. et al. Unprecedented electrocatalytic oxygen evolution performances by cobalt-incorporated molybdenum carbide microflowers with controlled charge re-distribution. *J. Mater. Chem. A* **9**, 1770-1783 (2021).
4. Zhang, Y. et al. Atomic iridium incorporated in cobalt hydroxide for efficient oxygen evolution catalysis in neutral electrolyte. *Adv. Mater.* **30**, 1707522 (2018).
5. Huang, Z.-F. et al. Chemical and structural origin of lattice oxygen oxidation in Co-Zn oxyhydroxide oxygen evolution electrocatalysts. *Nat. Energy* **4**, 329-338 (2019).
6. Zhao, S. et al. Ultrathin metal-organic framework nanosheets for electrocatalytic oxygen evolution. *Nat. Energy* **1**, 16184 (2016).
7. Wang, X. et al. Plasma-triggered synergy of exfoliation, phase transformation, and surface engineering in ccobalt diselenide for enhanced water oxidation. *Angew. Chem. Int. Ed.* **57**, 16421-16425 (2018).
8. Liardet, L. & Hu, X. Amorphous cobalt vanadium oxide as a highly active electrocatalyst for oxygen evolution. *ACS Catal.* **8**, 644-650 (2018).
9. Liu, W. et al. Boosting electrocatalytic activity of 3d-block metal (hydro)oxides by ligand-induced conversion. *Angew. Chem. Int. Ed.* **60**, 10614-10619 (2021).
10. Zhang, G. et al. Highly active and stable catalysts of phytic acid-derivative transition metal phosphides for full water splitting. *J. Am. Chem. Soc.* **138**, 14686-14693 (2016).
11. Zheng, X. et al. Multifunctional active-center-transferable platinum/lithium cobalt oxide heterostructured electrocatalysts towards superior water splitting. *Angew. Chem. Int. Ed.* **59**, 14533-14540 (2020).
12. Chen, L. et al. Facile synthesis of Cu doped cobalt hydroxide (Cu-$Co(OH)_2$) nano-sheets for efficient electrocatalytic oxygen evolution. *J. Mater. Chem. A* **5**, 22568-22575 (2017).
13. Han, X. et al. Ultrafine Pt nanoparticle-decorated pyrite-type CoS_2 nanosheet arrays coated on carbon cloth as a bifunctional electrode for overall water splitting. *Adv. Energy Mater.* **8**, 1800935 (2018).

14. Gong, L., Chng, X. Y. E., Du, Y., Xi, S. & Yeo B. S. Enhanced catalysis of the electrochemical oxygen evolution reaction by iron (III) ions adsorbed on amorphous cobalt oxide. *ACS Catal.* **8**, 807-814 (2018).
15. Yang, Z. et al. Trifunctional self-supporting cobalt-embedded carbon nanotube films for ORR, OER, and HER triggered by solid diffusion from bulk metal. *Adv. Mater.* **31**, 1808043 (2019).
16. Song, F. & Hu, X. Exfoliation of layered double hydroxides for enhanced oxygen evolution catalysis. *Nat. Commun.* **5**, 4477 (2014).
17. Zhang, B. et al. Homogeneously dispersed multimetal oxygen-evolving catalysts. *Science* **352**, 333-337 (2016).
